# An integrated strain-level analytic pipeline utilizing longitudinal metagenomic data

**DOI:** 10.1128/spectrum.01431-24

**Published:** 2024-09-23

**Authors:** Boyan Zhou, Chan Wang, Gregory Putzel, Jiyuan Hu, Menghan Liu, Fen Wu, Yu Chen, Alejandro Pironti, Huilin Li

**Affiliations:** 1 Division of Biostatistics, Department of Population Health, New York University School of Medicine, New York, New York, USA; 2 Department of Microbiology, New York University School of Medicine, New York, New York, USA; 3 Department of Biological Sciences, Columbia University in the City of New York, New York, New York, USA; 4 Division of Epidemiology, Department of Population Health, New York University School of Medicine, New York, New York, USA; Broad Institute, Cambridge, Massachusetts, USA

**Keywords:** microbiome, longitudinal metagenomic data, strain-level analysis, genomic variants, strain dynamics

## Abstract

With the development of sequencing technology and analytic tools, studying within-species variations enhances the understanding of microbial biological processes. Nevertheless, most existing methods designed for strain-level analysis lack the capability to concurrently assess both strain proportions and genome-wide single nucleotide variants (SNVs) across longitudinal metagenomic samples. In this study, we introduce LongStrain, an integrated pipeline for the analysis of large-scale metagenomic data from individuals with longitudinal or repeated samples. In LongStrain, we first utilize two efficient tools, Kraken2 and Bowtie2, for the taxonomic classification and alignment of sequencing reads, respectively. Subsequently, we propose to jointly model strain proportions and shared haplotypes across samples within individuals. This approach specifically targets tracking a primary strain and a secondary strain for each subject, providing their respective proportions and SNVs as output. With extensive simulation studies of a microbial community and single species, our results demonstrate that LongStrain is superior to two genotyping methods and two deconvolution methods across a majority of scenarios. Furthermore, we illustrate the potential applications of LongStrain in the real data analysis of The Environmental Determinants of Diabetes in the Young study and a gastric intestinal metaplasia microbiome study. In summary, the proposed analytic pipeline demonstrates marked statistical efficiency over the same type of methods and has great potential in understanding the genomic variants and dynamic changes at strain level. LongStrain and its tutorial are freely available online at https://github.com/BoyanZhou/LongStrain.

**IMPORTANCE:**

The advancement in DNA-sequencing technology has enabled the high-resolution identification of microorganisms in microbial communities. Since different microbial strains within species may contain extreme phenotypic variability (e.g., nutrition metabolism, antibiotic resistance, and pathogen virulence), investigating within-species variations holds great scientific promise in understanding the underlying mechanism of microbial biological processes. To fully utilize the shared genomic variants across longitudinal metagenomics samples collected in microbiome studies, we develop an integrated analytic pipeline (LongStrain) for longitudinal metagenomics data. It concurrently leverages the information on proportions of mapped reads for individual strains and genome-wide SNVs to enhance the efficiency and accuracy of strain identification. Our method helps to understand strains’ dynamic changes and their association with genome-wide variants. Given the fast-growing longitudinal studies of microbial communities, LongStrain which streamlines analyses of large-scale raw sequencing data should be of great value in microbiome research communities.

## INTRODUCTION

With the steady growth of longitudinal microbiome studies, microbiome is now on the cusp of clinical utility for several diseases, including obesity (1, 2), diabetes (3, 4), inflammatory bowel disease (5, 6), and cancer (7, 8). The characterization of microbiome was conventionally constrained to taxonomic classifications of 16S rRNA sequences (9). However, recent advancements in metagenomic sequencing technology and analytic tools have facilitated precise interpretations of the microbial profile and functional attributes at the species or even strain level (10). While species-level identification typically offers adequate resolution in the analysis of metagenomic sequencing data, it is essential to recognize that different strains within a species may exhibit substantial phenotypic variability (11). Until now, investigations at the strain level have uncovered extensive diversity within an individual’s microbiome (12), which has substantial implications for understanding antibiotic resistance (13) and pathogen virulence (14). Furthermore, studies have revealed the coexistence of multiple lineages and signals of within-patient selection in specific microorganisms, such as *Burkholderia dolosa* (15) and *Staphylococcus epidermidis* (16). Hence, investigating within-species variation holds significant promise in understanding the molecular mechanism of microbial biological processes.

There are basically two distinct but complementary categories of strain-level analytic tools, i.e., metagenome-assembled approaches and reference genome-based approaches (11). Metagenome-assembled approaches, such as STRONG (17), recover genome sequences directly from shotgun metagenomic sequencing data. Metagenome-assembled genomes, however, may embody a composite of biological haplotypes within the sampled population (18, 19) and are constrained by the assembly quality and incompleteness (11). In reference genome-based strategies, shotgun-sequencing reads are typically aligned to pre-defined marker genes or species-reference genomes for the derivation of genotyping outcomes, as exemplified by tools like StrainPhlAn (20), metaSNV (21), MIDAS (22), inStrain (23), and GT-Pro (24). Additionally, various methodologies have been devised to enable strain deconvolution by harnessing multiple metagenotypes (e.g., a collection of longitudinal samples), including Lineage (25), ConStrains (26), DESMAN (27), StrainFinder (28), StrainFacts (29), and StrainGE (30). There are also many additional tools developed for various study designs and clinical applications, which have been well reviewed (31). However, most genotyping tools do not encompass strain deconvolution, while strain-deconvolution methods based on allele frequencies inadequately exploit the genome-wide single nucleotide variants (SNVs) and haplotype information contained within sequencing reads.

Identical strains of a species are often shared across single-patient microbiome samples that are obtained from different body sites or over time (12, 32). In such instances, our interest extends beyond SNVs solely in dominant strains, defined as strains with within-species proportion (*P*) ≥50% at individual time points (20), to encompass genomic variants in non-dominant strains and the dynamic changes in their proportions. Thus, it is critical to jointly model the proportions and genetic variants of strains in longitudinal samples by utilizing raw sequencing data. There are multiple merits in doing this. Firstly, this allows us to more accurately detect the variants of the dominant strain and non-dominant strain (if present). Some methods mainly focus on a dominant strain or major alleles (20). For example, Garud’s pipeline (33) built on MIDAS required quasi-phaseable (QP) samples with *P* > 80% in the core genome, which neglects a large number of non-QP samples. When two strains have comparable proportions, it is challenging to assign variants correctly to the strains. However, if multiple samples from one subject are available, allele information from other samples can be leveraged to phase the SNVs between the dominant and the non-dominant strains (i.e., strain deconvolution). Secondly, variants that are assigned correctly to each strain will benefit the estimation of strain proportion in return, as genome-wide variants can greatly improve the accuracy of strain proportions compared to the one estimated based on marker genes. Thirdly, we can monitor the dynamic change of strains by modeling strain transitions within species using longitudinal samples (32, 34, 35).

In this paper, we developed LongStrain, a reference genome-based microbial community profiling pipeline designed for longitudinal metagenomic data. LongStrain detects genome-wide SNVs and estimates strain proportions. For each target species within a subject, the pipeline generates outputs that include genome-wide SNVs in variant call format for the two most abundant strains, along with their proportion estimates across time points. To validate our approach, we conducted extensive simulations comparing LongStrain with four widely used tools, including two genotyping methods (StrainPhlAn4 and MIDAS2) and two deconvolution methods (ConStrains and DESMAN). Additionally, we tested LongStrain on a subset of The Environmental Determinants of Diabetes in the Young (TEDDY) study (34) and a study of oral and gastric microbiome in relation to gastric intestinal metaplasia (36).

## RESULTS

### Strain-level analytic pipeline for longitudinal metagenomic data

**Fig 1:**
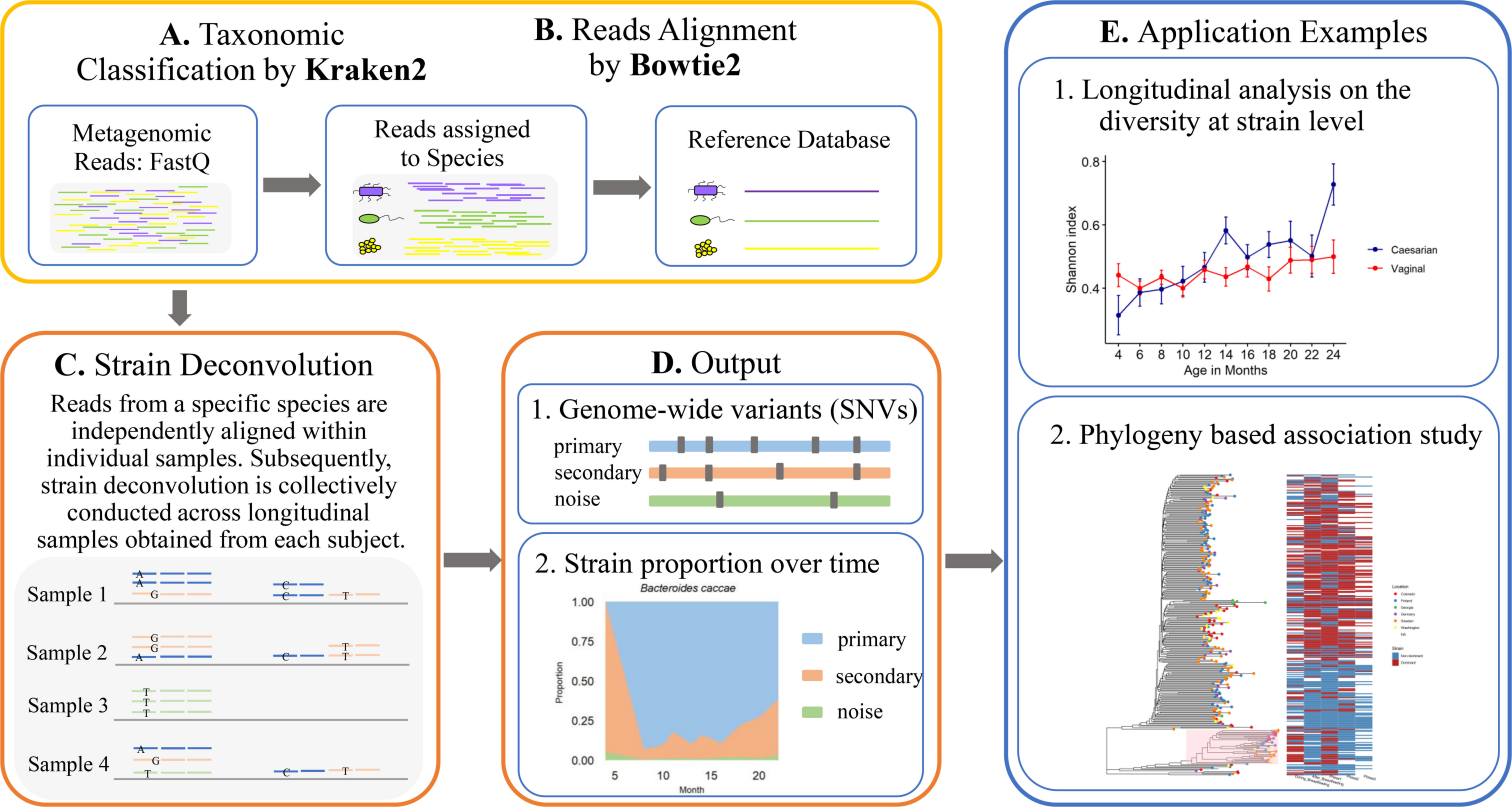
The LongStrain pipeline. (A) Raw reads are assigned at species level by Kraken2. (B) Assigned reads are aligned to the representative genome of each species from the database. (C) Within each species, SNVs and strain proportions are inferred jointly by our algorithm in longitudinal or concurrent samples. (D) LongStrain outputs SNVs and proportions of the primary and secondary strains in longitudinal or concurrent samples. (E) Examples illustrating the application of LongStrain’s output.

LongStrain is an integrated pipeline for detecting genomic variants at strain level using metagenomic data from individuals with longitudinal or concurrent samples (Fig. 1). The entire workflow consists of two core components: “Reads Assignment,” which relies on Kraken2 (Fig. 1A) and Bowtie2 (Fig. 1B), and “Strain Deconvolution” (Fig. 1C). Within each subject, raw reads from multiple samples are first assigned to each species by Kraken2 (37), which is an efficient tool for taxonomic classification of metagenomic data with both high precision and sensitivity (38, 39). Subsequently, reads assigned to species level are mapped to the reference genomes from the database by Bowtie2 (40). The Genome Taxonomy Database (GTDB) is a repository characterized by a standardized taxonomy, wherein a representative genome is provided for each species. It is constructed by organizing all genomes from NCBI Assembly database into species clusters based on the average nucleotide identity (ANI). Within our pipeline, we have integrated both the GTDB and the NCBI database, affording users the flexible transition between the two repositories through a straightforward command-line option. Then, the alignment files generated by Bowtie2 are inputted into our strain deconvolution algorithm.

In longitudinal investigations of host microbiome, a single strain commonly dominated each species and was retained over time within individual subjects (20, 33). Nevertheless, in some cases, the initial dominant strain may be replaced by a non-dominant strain or a new strain coming into the community. In order to model the possible transition of the dominant strains at certain time point, our algorithm focuses on the two most abundant strains (namely a primary strain and a secondary strain) within subjects for each species. In this study, we delineate the “primary” strain as the one exhibiting the highest proportion of reads for a given species within a subject and, correspondingly, designate the “secondary” strain as the strain with the second-highest proportion, with any remaining strains considered as the “noise” strain. It is worth noting that these definitions are made upon aggregating all longitudinal samples from a subject, distinguishing from the dominant/non-dominant strains established by within-species proportion (*P*) at individual time points.

For each given species within individual subjects, LongStrain screens the whole genome of the species to identify strains’ SNVs and construct haplotypes. Due to the low mutation rate of DNA-based genomes (41), the haplotypes within the same strain across multiple samples from one subject are assumed to be consistent as in most other methods (26, 27). In other words, *de novo* mutations occurring within the timespan of the longitudinal study are not incorporated into our algorithm because of their rarity compared to the total number of SNVs. After obtaining sequence alignment files, the sequencing reads for each species within samples are piled up using Pysam (42), as illustrated in Fig. 1C. Subsequently, the piled reads and associated SNVs are phased into the corresponding strains according to our maximum likelihood model (see Materials and Methods for details of the strain-deconvolution algorithm), and variants that cannot be classified are regarded as being from the noise strain.

### Performance assessment using simulated microbial communities (Gut20)

We simulated a human gut microbial community consisting of 20 species mostly from the Gut20 scenario in Ounit’s study (43, 44). For each species, raw sequencing reads were (at depth of 10×) simulated from two reference genomes to mimic primary and secondary strains, and multiple scenarios of longitudinal fluctuations of strain compositions were designed at three time points (Materials and Methods). At each time point, the generated reads of all 20 species were pooled to obtain the simulated samples of a microbial community. The performance evaluation of four methodologies for SNV identification was conducted through precision and recall metrics, as outlined in the Materials and Methods section. ConStrains was excluded from this comparative analysis due to its reliance on the marker gene database in MetaPhlAn, which does not allow the customization of database. In the assessment of strain proportions, LongStrain was exclusively compared with ConStrains and DESMAN, as the remaining two tools did not furnish this particular outcome.

**Fig 2:**
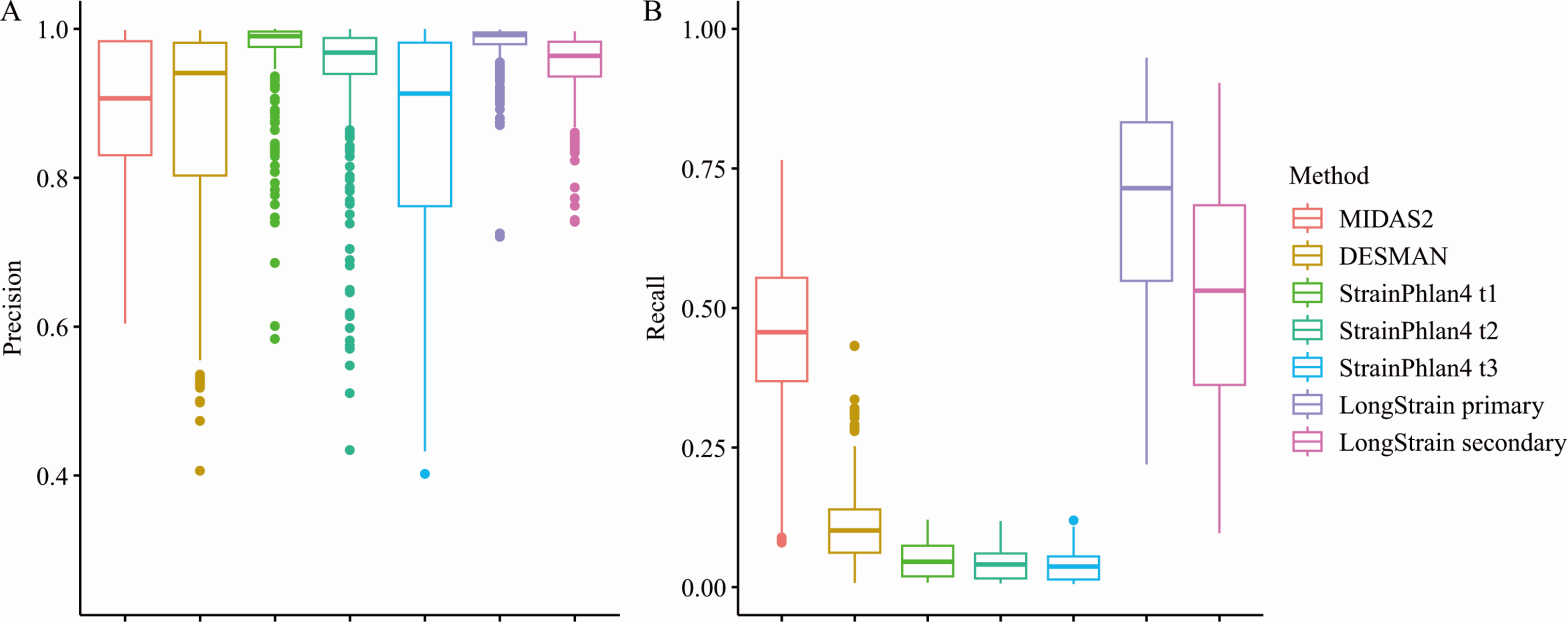
Comparison of LongStrain, MIDAS2, DESMAN, and StrainPhlAn4 in community simulation across 20 repetitions. The gut microbial community (Gut20) consists of 20 species from 15 genera. For each species, the reference genomes of two strains are randomly selected from the GTDB database. The raw reads of each species are simulated at three time points according to various scenarios with a modest sequencing depth of 10×. (A) Precision of SNV calling by these methods; (B) recall of SNV calling by these methods.

The performance of the methods was evaluated through 20 repetitions of the simulation procedure. Across the simulated community comprising 20 species, LongStrain consistently identified all 20 species in each iteration, whereas MIDAS2 reported 19 species, and both DESMAN and StrainPhlAn4 reported 17 species in their respective analyses. The precision and recall distributions for all species across 20 repetitions were depicted in Fig. 2A and B, and detailed results for specific species were additionally compiled in Table S1 and S2. Notably, the primary strain identified by LongStrain exhibited the highest precision, closely rivaling that reported by StrainPhlAn4 at the initial time points (Fig. 2A). Although the precision of the second strain identified by LongStrain is slightly lower, it surpassed that of MIDAS2 and DESMAN. Furthermore, both the primary and secondary strains identified by LongStrain demonstrate higher recall rates for SNVs compared to those identified by other methodologies (Fig. 2B). Following closely behind LongStrain, MIDAS2 exhibited higher recall than the other methods. With its constrained marker-based database, StrainPhlAn4 unsurprisingly had the lowest recall.

Regarding estimation of strain proportions, we compared the primary-strain proportions estimated by LongStrain with ConStrains and DESMAN at three time points. Since there were only two strains simulated in the data, the result was regarded as an unsuccessful estimation if more than three strains were estimated in ConStrains. On the simulated data set and averaged across 20 repetitions, ConStrains only reported the proportions of strains in 16 out of the 20 species, while DESMAN and LongStrain reported 17 and 20 species, respectively (Table 1). We measured the accuracy of the estimated proportions by mean absolute error (MAE) and root mean square error (RMSE) at three time points in each species (Materials and Methods). LongStrain had higher accuracy than the other two methods in 18 out of 20 species (Table 1). In summary, LongStrain exhibited better overall performance compared to the other four methods across the majority of the 20 species in our community simulation.

**TABLE 1:** The accuracy of the estimated proportion of the primary strain by ConStrains, DESMAN, and LongStrain^*a*^

Species	ConStrains			DESMAN				LongStrain	
	*N*	MAE	RMSE	*N*	MAE	RMSE	*N*	MAE	RMSE
*Acinetobacter baumannii*	20	0.054	0.067	20	0.058	0.076	**20**	**0.015**	**0.019**
*Bacillus cereus*	–	–	–	**20**	**0.068**	**0.089**	20	0.105	0.115
*Bacteroides fragilis*	18	0.079	0.092	20	0.099	0.133	**20**	**0.023**	**0.034**
*Bifidobacterium adolescentis*	14	0.073	0.082	20	0.085	0.11	**20**	**0.025**	**0.033**
*Bifidobacterium bifidum*	19	0.047	0.053	20	0.088	0.115	**20**	**0.02**	**0.027**
*Bifidobacterium breve*	16	0.05	0.057	20	0.093	0.112	**20**	**0.032**	**0.043**
*Bifidobacterium longum*	17	0.057	0.066	20	0.107	0.134	**20**	**0.021**	**0.023**
*Clostridium beijerinckii*	8	0.275	0.298	–	–	–	**20**	**0.029**	**0.036**
*Enterococcus faecalis*	18	0.066	0.075	–	–	–	**20**	**0.018**	**0.026**
*Escherichia coli*	14	0.074	0.086	20	0.192	0.215	**20**	**0.053**	**0.06**
*Helicobacter pylori*	17	0.064	0.073	–	–	–	**20**	**0.05**	**0.059**
*Lactobacillus gasseri*	12	0.093	0.11	20	0.076	0.084	**20**	**0.022**	**0.035**
*Listeria monocytogenes*	17	0.095	0.113	20	0.093	0.114	**20**	**0.026**	**0.036**
*Neisseria meningitidis*	16	0.067	0.082	20	0.049	0.067	**20**	**0.032**	**0.036**
*Pseudomonas aeruginosa*	19	0.051	0.067	20	0.037	0.045	**20**	**0.012**	**0.018**
*Rhodobacter sphaeroides*	18	0.051	0.071	20	0.294	0.302	**20**	**0.015**	**0.022**
*Staphylococcus aureus*	19	0.05	0.066	20	0.122	0.163	**20**	**0.016**	**0.022**
*S. epidermidis*	17	0.072	0.087	20	0.189	0.229	**20**	**0.069**	**0.08**
*Streptococcus agalactiae*	15	**0.065**	**0.075**	20	0.249	0.267	20	0.206	0.226
*Streptococcus mutans*	17	0.068	0.081	20	0.074	0.09	**20**	**0.025**	**0.034**

^*a*^ *N*, the number of valid reports across 20 repetitions of the simulation. “-” means no valid result for th species. Values in bold highlight the better performing method for a given species.

### Performance assessment using simulated reads from single species

The performances of these methods are potentially influenced by other factors: sequencing depth, the scenarios depicting longitudinal fluctuations in strain compositions, and ANIs of strains relative to the representative genome. In this section, we specifically delve into the examination of these factors by focusing on a particular microbial species, namely *Bifidobacterium breve*. We firstly investigate two illustrative temporal scenarios to model longitudinal fluctuations in strain compositions. The first scenario ([9:1, 6:4, 3:7]) represents a transitional period, wherein the ratio of sequencing depths between the primary strain and the secondary strain evolves from 9×:1× at the initial time point to 6×:4× and 3×:7× at two subsequent time points. In the second stable scenario ([8:2, 8:2, 8:2]), the ratio of sequencing depths for the two strains remains constant at 8×:2× across three consecutive time points.

We conducted a comparative analysis of precision (Table S3) and recall (Table S4) for four methods as in the community simulation at average depths of 5×, 10×, and 20× in both transitional and stable scenarios. As the average depth increased, all methods exhibited improved performance as anticipated. At the depth of 5×, MIDAS2 and DESMAN did not generate valid results in either scenario. At the depth of 10× and 20×, MIDAS2 consistently demonstrated higher precision in stable scenarios compared to transitional scenarios. These results suggest that treating the consensus alleles as a real strain may be inappropriate in practical application, except when all allele frequencies are close to one. In contrast, DESMAN exhibited higher precision and recall in transitional scenarios as opposed to stable scenarios. Given its utilization of a set of marker genes, StrainPhlAn4 consistently achieved low recalls and exhibited slightly increased precisions with escalating sequencing depth, particularly demonstrating higher precisions in stable scenarios. Notably, LongStrain achieved the highest levels of recall (~99.3%) and precision (~74.1%) for the primary strain across varying sequencing depths, irrespective of the scenario. For the secondary strain, LongStrain showcased higher precisions in transitional scenarios (~98.7%) compared to stable scenarios (~95.3%). It also showed higher recalls compared to primary strains analyzed by other methods, except for MIDAS2 in the stable scenario at the sequencing depth of 20×.

In terms of the bias of the estimated proportions, LongStrain had better performance than DESMAN and ConStrains in nearly all instances, with the exception being the transitional scenario at a sequencing depth of 20×, wherein DESMAN exhibited the lowest bias (Table S5). At a depth of 5×, DESMAN and ConStrains failed to produce valid results. In contrast, LongStrain demonstrated reduced sensitivity to sequencing depth and scenario variations, maintaining an MAE of less than 2% in all circumstances. Similarly, aligning with the trends observed in precision and recall, DESMAN manifested superior performance in the transitional scenario compared to the stable scenario.

In summary, LongStrain outperformed the other four competing methods in most situations, particularly at relatively low sequencing depths (5 ~ 10×). MIDAS2 and StrainPhlAn4, not designed as strain-deconvolution methods, exhibited enhanced performance under stable scenarios as opposed to transitional scenarios, which was a distinction from DESMAN. As sequencing depth increased, LongStrain consistently yielded comparable results to alternative methods in both SNV calling and estimation of strain proportion, regardless of the underlying scenario.

In investigating the impact of strains’ ANIs relative to the representative genome, we created ANI gradients (ranging from 99% to 97%) by introducing random mutations to the original reference genomes of *B. breve*. Subsequently, we assessed the performance of all methods under both transitional and stable scenarios, employing a modest sequencing depth of 10×. As illustrated in Tables S6 to S8, the performances of all methods generally were consistent with those observed using the original reference genomes. These results also showed the robustness of these methods to the decreasing of ANI. Notably, LongStrain’s performance exhibited minimal degradation with decreasing ANIs, consistent with its underlying algorithm. This alignment stemmed from LongStrain’s ability to leverage a greater number of variants for strain deconvolution with decreasing ANIs.

Despite our method being designed for situations involving only two strains, we evaluated its performance in scenarios with three strains at a sequencing depth of 10×, where noise strains constituted non-negligible proportions. Building on the transitional and stable scenarios mentioned earlier, we introduced 10% noise strains at each time point to create the scenarios [8:1:1, 5:4:1, 2:7:1] and [7:2:1, 7:2:1, 7:2:1]. Additionally, we devised two scenarios involving three strains ([9:1:0, 6:0:4, 3:7:0] and [8:2:0, 8:0:2, 0:8:2]), each with no more than two strains per sample. As demonstrated in the previous analysis, we presented the precision and recall results in Fig. S1 and summarized the bias in estimated proportions in Table S9. Consistent with the earlier results, DESMAN exhibited superior performance in transitional scenarios compared to the stable scenario [7:2:1, 7:2:1, 7:2:1], whereas MIDAS2 demonstrated the second-highest recall and performed better in stable scenarios than in transitional scenarios. LongStrain’s primary strain maintained the highest precision and recall levels compared to other methods. However, the precision of LongStrain’s secondary strain decreased to around 90%, suggesting that only the results for the primary strain by LongStrain are valid when the noise strain is non-negligible. Regarding the bias in estimated strain proportions, LongStrain outperformed ConStrains except in the scenario [8:2:0, 8:0:2, 0:8:2], while DESMAN had the best performance except in the stable scenario [7:2:1, 7:2:1, 7:2:1].

### Using LongStrain to obtain further insights from previous microbiome studies

We applied LongStrain to two data sets from previously reported microbiome studies. The first data set stems from TEDDY study, and we use it to showcase four major applications of LongStrain in metagenomics research. LongStrain can (i) provide longitudinal proportion estimates of strains within species, which enable temporal pattern analysis at strain level; (ii) identify strain transitions, which allows strain transition time to be compared to other time-dependent factors; (iii) study diversity at the strain level; and (iv) conduct phylogenetic tree-based strain-level association tests, which enable the identification of strains or species subclades related to the relevant clinical outcome. The second real data comes from a study of the oral and gastric microbiome in relation to gastric intestinal metaplasia, with which we add another interesting example of phylogenetic tree-based strain-level association tests (the fourth application in the above list).

### Real data 1: TEDDY metagenomics study

TEDDY study contains longitudinal metagenomics sequencing of stool samples from 783 infants, including 415 controls, 267 seroconverters, and 101 individuals diagnosed with Type 1 diabetes (T1D) (34). These samples were collected quarterly from 3 to 46 months of age, presenting a unique opportunity to investigate the development of microbiome during infancy and childhood, as well as to identify the associated external factors. We first retrieved a set of 100 subjects with T1D diagnosis and 100 controls from TEDDY project (34) including six clinical research centers in the USA (Colorado, Georgia/Florida, and Washington) and Europe (Finland, Germany, and Sweden). We applied LongStrain to the data set and screened 44 species (Supplemental File) with relatively high abundance reported in two previous TEDDY studies (34, 45). Ultimately, 26 species, meeting the criteria of adequate sequencing depth (i.e., total genome-wide vertical coverage >10× in longitudinal samples) in at least 20 subjects, were retained for subsequent strain-level analysis. For most studied species, there was a strain with *P* > 80% in TEDDY samples (Fig. S2), which agrees with previous studies (20, 33). However, there was still a considerable proportion (ranging from 15.2% to 67.4%) of samples in which the highest *P* was ≤80% for these 26 species, which emphasizes the necessity to take non-QP samples into account.

### Application 1: longitudinal analysis of strain proportion

**Fig 3:**
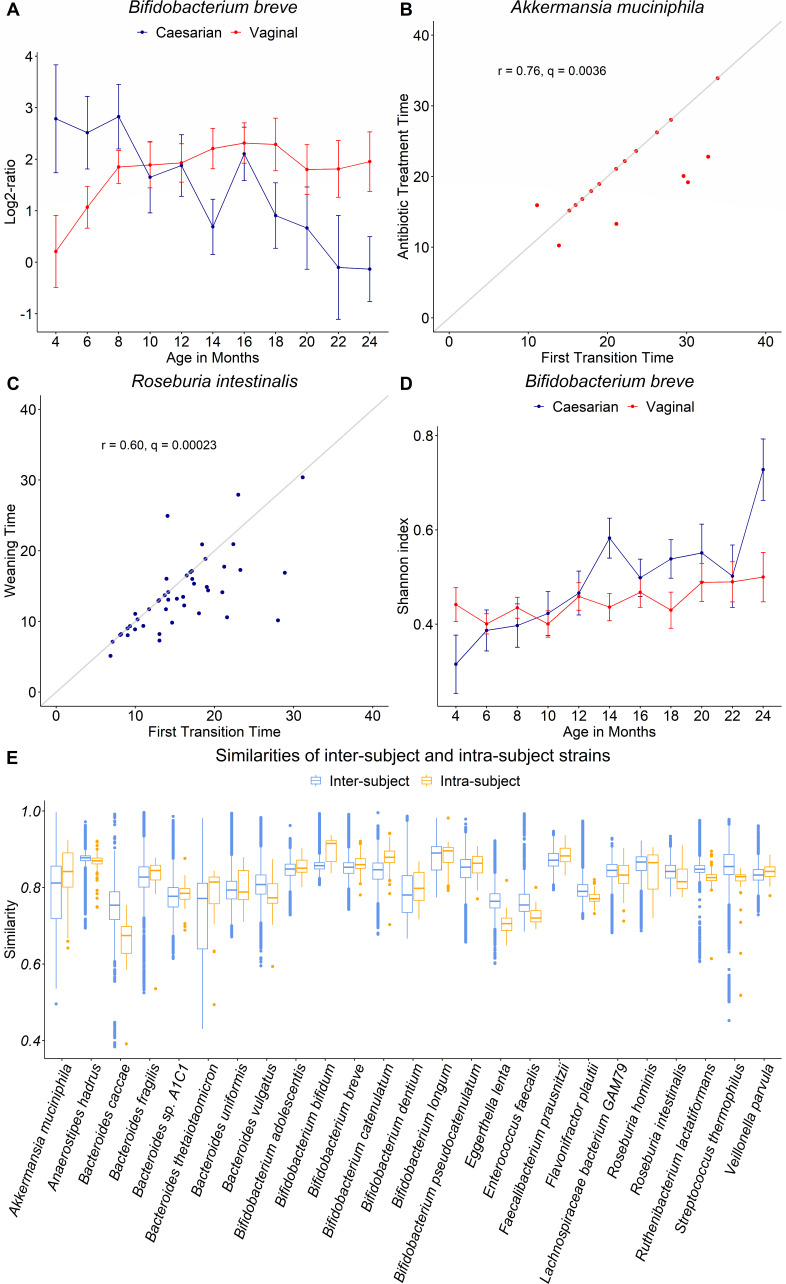
(A) Log2-ratio of the proportions of the primary strain to the secondary strain by birth delivery mode in *B. breve*. (B) Scatter plot and Pearson correlation test of time (in months) of the first transition and the antibiotic treatment in *A. muciniphila*. (C) Scatter plot and Pearson correlation test of time (in months) of the first transition and the weaning in *R. intestinalis*. (D) Shannon index of the strains by different delivery modes within *B. breve*. (E) Similarity of inter-subject and intra-subject strains in 25 species.

We wanted to first examine the complex interplay of strains within a species, and identify the associated external factors which contribute to shaping the microbial composition of the gut during early childhood (32). Implementing LongStrain on the subset of TEDDY study, we obtained the proportion estimates of the two most abundant strains for each species at each time point within subjects. We then calculated the log2-ratio of the proportions of the primary strain to the secondary strain, and its sign indicated which strain was more abundant at each sample. The log2 transformation helped to normalize the data, preparing for the following linear mixed effect model (LMM). We then fit the log2-ratios in the linear mixed effect model to check their longitudinal trend with the infant’s delivery mode, while adjusting for sex (as described in the Materials and Methods section). In Table S10, we reported five species in which the temporal trends of the log2-ratio were significantly different between two birth delivery modes after false discovery rate (FDR) correction: *B. breve* (*q* = 5.79E − 08), *Akkermansia muciniphila* (*q* = 0.020), *Bacteroides thetaiotaomicron* (*q* = 0.020), *Faecalibacterium prausnitzii* (*q* = 0.034), and *Roseburia intestinalis* (*q* = 0.034). As depicted in Fig. 3A, infants born via vaginal delivery displayed a log2-ratio starting at around 0, indicating a balance between two strains, and then increasing as they aged. This gradual increase suggests that one strain gradually became more abundant than the other. Conversely, infants born via cesarean initially displayed a log2-ratio starting at a positive value, indicating that one *B. breve* strain was more abundant than the other, followed by a decrease as they grew older, with the value shifting from positive to negative. These observations suggest a strain transition, with the strain initially present in a smaller proportion transitioning to become the more abundant strain around the 22nd month, at which point the log2-ratio equaled approximately zero. In the next application, we will delve deeper into the analysis of strain transition.

### Application 2: identifying strain transitions

If a dominant strain (*P* ≥ 50%) shifts to a non-dominant status (*P* < 50%) within longitudinal samples of a subject, we term this occurrence a “strain transition,” potentially signifying a significant shift in the local microenvironment. With the above definitions, we investigated whether a transition happened in subjects for the 26 species we studied in “Application 1”. We found that strain transitions were prevalent in most species in the early gut microbiome (months 3 to 46) (Fig. S3). The proportion of subjects with a strain transition ranges from 18.4% (in *Bacteroides thetaiotaomicron*) to 96% (in *Lachnospiraceae bacterium GAM79*) in 26 species.

Next, we focused on the subjects who had early life events (antibiotic treatments or weaning) and at least one strain transition to explore their relationship. We performed the Pearson correlation test and drew the scatter plot of the timing of the first transition and early life event. After FDR correction, we observed significant correlations between the timing of the first strain transition and the timing of the first use of antibiotics in *A. muciniphila* (*r* = 0.76, *q* = 0.0036), *Bifidobacterium bifidum* (*r* = 0.69, *q* = 0.0036), *Bifidobacterium longum* (*r* = 0.40, *q* = 0.0072), and *E. coli* (*r* = 0.48, *q* = 0.0036) (Fig. 3B; Fig. S4A through C). Particularly in *A. muciniphila*, the first strain transitions coincided with the antibiotic treatments in subjects, which was indicated by the points on the diagonal line (Fig. 3B). Weaning was found to be correlated with the first strain transition in *R. intestinalis* (*r* = 0.60, *q* = 0.00023), *B. bifidum* (*r* = 0.54, *q* = 0.0021), *B. longum* (*r* = 0.42, *q* = 0.00086), and *Roseburia hominis* (*r* = 0.71, *q* = 0.0031) (Fig. 3C; Fig. S4D through F).

### Application 3: analysis of intra- or inter-subject diversity at strain level

With the proportions of strains outputted from LongStrain, we calculated the Shannon’s index of strain proportions within species to measure the strain diversity (Materials and Methods). We also fit LMMs to test whether the temporal trend of Shannon’s diversity over time was associated with two birth delivery modes (Materials and Methods). A significant association was only observed in *B. breve* (*q* = 0.0072) after adjusting for sex. Vaginally delivered infants has a higher temporal variation of strain-level diversity in *B. breve* than cesarean-born infants (Fig. 3D), which is consistent with the delayed colonization of *B. breve* in C-section-delivered infants (46).

In this study, strain similarity between two strains is quantified as the percentage of identical genotypes at polymorphic sites across all samples. We summarized the similarities by intra- and inter-subject strains in 25 species (*E. coli* was omitted due to its low horizontal coverage, Fig. 3E). The former was the similarity between the two most abundant strains within each subject and the latter was the similarity between strains across different subjects. The median inter-subject similarity in most species ranged from 70% to 90%, and the median intra-subject similarity is close to that of inter-subject similarity in most species, which indicates that two strains within a subject do not tend to have a higher strain similarity than strains from different subjects.

We were interested in investigating whether the strain similarity was related to the case-control status. Specifically, if the strains within cases or within controls are genetically more uniform, this should manifest as a higher strain similarity within either of the groups. To test this hypothesis, we first calculated the similarities between the pairs of strains in three groups: “*case vs case*,” “*control vs control,*” and “*case vs control*”; then, we compared the distributions of similarity values within each group. However, we did not find significantly different distributions of similarity between “*case vs case*” and “*control vs control*” groups after screening 26 species above. To eliminate the effect of the primary or secondary strain, the above analysis was repeated within pairs of primary strains and pairs of secondary strains, respectively, and the conclusion did not change. As illustration examples, the results of *A. muciniphila*, *Bacteroides uniformis*, and *B. longum* are shown in Fig. S5 to S7. It may imply that the onset of T1D is not associated with specific phylogenetic clusters of strains for these species.

### Application 4: LongStrain in phylogenetic tree-based strain-level association test

**Fig 4:**
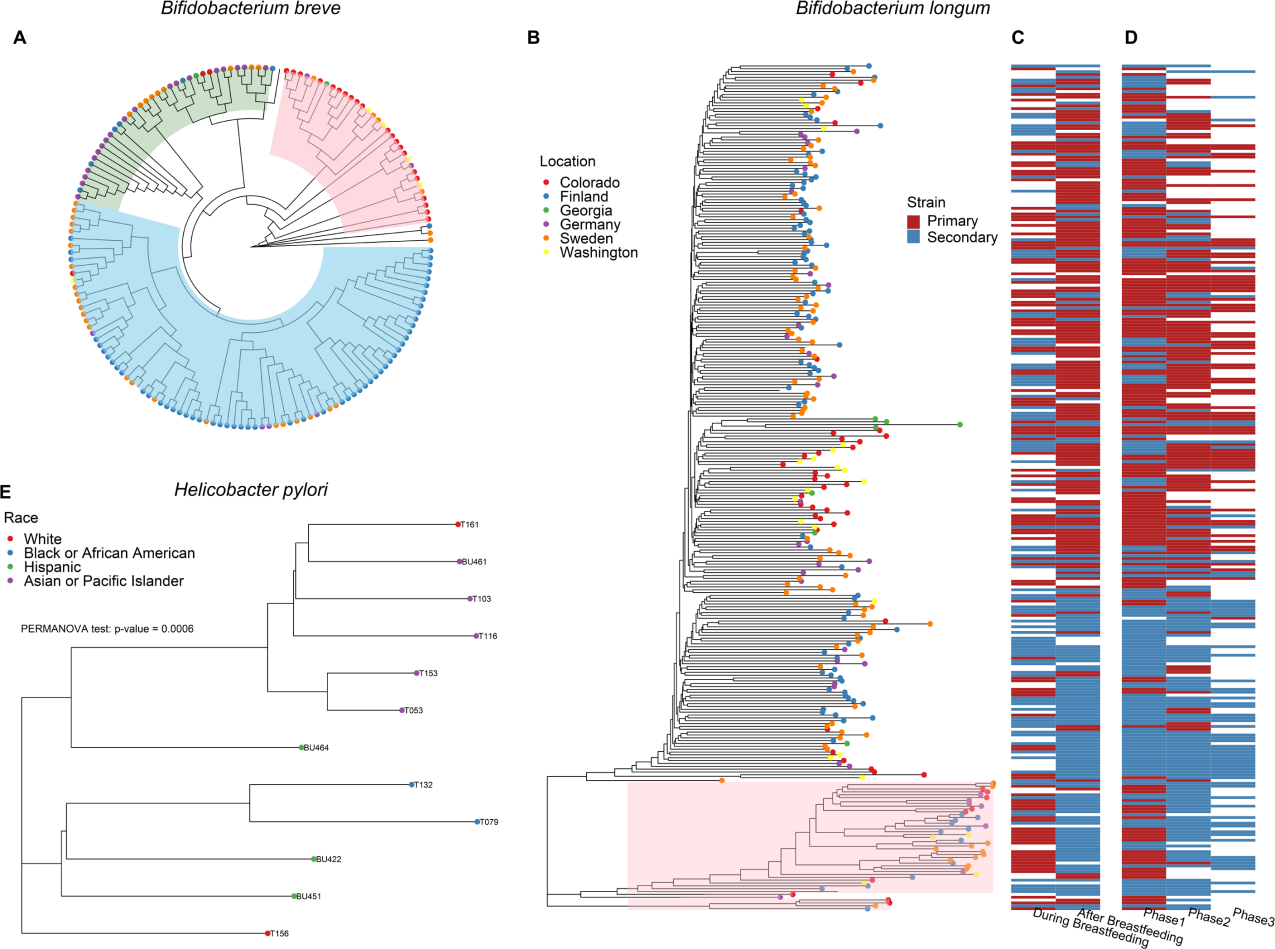
(A) Phylogenetic tree of strains in *B. breve* from six centers. Highlighted in pink: mostly from three centers in the USA; highlighted in blue: mostly from two Northern European countries (Finland and Sweden); highlighted in green: diversified sources from Europe. (B) The phylogenetic tree of *B. longum* in samples with T1D diagnosis from TEDDY study. The sources of strains are marked by colored dots. The subclades highlighted in pink are associated with breastfeeding and phases of microbiome progression. (C) The status (primary or secondary) of strain before and after breastfeeding. (D) The status (primary or secondary) of strain in phase 1 (months 3–14), phase 2 (months 15–30), and phase 3 (≥31 months). (E) Phylogenetic tree of primary strains in *Helicobacter pylori* from 12 subjects.

LongStrain also produced genomic variants derived from the species identified in TEDDY study, with which we constructed the neighbor-joining trees within species by MEGA-X (47), and calculated the phylogenetic distances between strains. We then investigated its association with the host phenotypes. By labeling the geographical information of the samples on the tree, we would like to explore the relationship between the phylogenetic structure of microbes and geographically separated host populations. This biogeographical pattern is primarily shaped by host migrations and microbial transmission mechanisms (20). Given that TEDDY cohort predominantly comprised non-Hispanic white individuals, the strains within most species displayed no distinct population structure within Westernized populations. Specifically, most species did not show discrete population structure within Westernized populations (e.g. Fig. S8), in accordance with a previous study (20). Nonetheless, we observed some clusters of strains by geographic location in *B. breve* and *B. longum*. Particularly in *B. breve* (Fig. 4A), strains highlighted in pink were almost exclusively from three centers (Colorado, Georgia, and Washington) in the USA and subclades highlighted in blue mostly consisted of strains from two Northern European countries (Finland and Sweden). Conversely, subclades in green showed diversified sources of strains. Additionally, similar patterns were observed in the phylogenetic tree of *B. longum* (Fig. 4B). Although not as distinct as those in *B. breve*, some strains from the USA or Northern European countries clustered under some subclades of *B. longum*. This result indicates a predominance of vertical transmission in these two species, consistent with findings from prior studies (48, 49).

To further explore whether subclades were correlated with other factors, we tested the association between the phylogenetic patterns of strains and T1D diagnosis, delivery mode, antibiotics, breastfeeding, and phases of microbiome progression separately. Intriguingly, we observed that only a subspecies clade of *B. longum* was associated with breastfeeding or phases of microbiome progression (Fig. 4C). The status (primary or secondary) of each strain was recalculated before and after breastfeeding. The majority of the subspecies clade highlighted in pink (Fig. 4B and C) displayed a switch from “primary” to “secondary”. The microbiome progression in early life was previously defined as three phases, a developmental phase (months 3–14), a transitional phase (months 15–30), and a stable phase (≥31 months) according to the Shannon’s diversity index (45). The status (primary or secondary) of each strain was recalculated in each phase. Naturally, a similar transition of strain status was observed from the first phase to the third phase for this clade (Fig. 4D). This phylogenetic pattern coincided with the well-characterized subspecies clade of *B. longum* and *Bifidobacterium infantis*, identified in the DIABIMMUNE study in Finland, Estonia, and Russian Karelia (35). *B. infantis* is capable of efficiently consuming several small mass human milk oligosaccharides (HMOs) and harbors a wide variety of genes dedicated to HMO metabolism (50, 51), which explains its dominance during breastfeeding (or the first phase) and dissipation over time. This analysis highlights that the use of LongStrain empowers us to identify subclades with distinct functions in longitudinal metagenomics data.

### Real data 2: a gastric intestinal metaplasia microbiome study (example for Application 4)

We retrieved oral and gastric microbiome data from a case-control study including 89 cases with gastric intestinal metaplasia (IM) and 55 matched controls in New York City, USA (36). Oral wash and antral mucosal brushing samples from the same subject were treated as repeated measures in this analysis.

Although *H. pylori*-induced gastritis plays an essential role in gastric cancer initiation, only about 3% infected individuals develop malignancy (52, 53). Therefore, in this analysis, we focused on *H. pylori* and sought to identify strains or subclades of *H. pylori* closely related to gastric IM. Unfortunately, due to the fading of *H. pylori* under achlorhydric condition in precancerous lesions (54), *H. pylori* was only detected in 12 subjects with enough sequencing depth (>10×). Since there was no secondary strain with enough genome coverage (depth <1×), we focused on the primary strains from these subjects in the phylogenetic analysis. Despite the small sample size, the phylogenetic distances between strains were significantly associated with the race of the hosts by permutational multivariate analysis of variance (PERMANOVA, *P*-value <0.001, Fig. 4E). We observed the clustering of strains from African Americans and Asians, while not in Hispanics or Whites. This may be attributed to the cohesion of community or the dietary habits in these two ethnic groups. On the other hand, the evidence of relatedness between strains and gastric IM was not found in the phylogenetic analysis. For further investigation of the association between gastric cancer and variants in *H. pylori*, more genomes of *H. pylori* with high sequencing depth are needed.

## DISCUSSION

In this study, we introduced an integrated pipeline for the genome-wide variant calling and strain proportions estimation in longitudinal metagenomic data. By comparing LongStrain with four popular tools on the simulated data sets, we demonstrated that LongStrain is superior or comparable to these methods in both accuracy and reporting rate under various scenarios. In the real data analysis, we applied LongStrain to a subset of TEDDY project to extensively characterize prevalent species at strain level. Our results are in agreement with previous observations that there is a strain with *P* > 80% within most species for most samples (20). We also observed prevalent transitions of the dominant strain in some species during the early life of infants, which may be associated with events like antibiotic treatments and dietary changes. We phylogenetically profiled the strains in more than 20 species with the SNVs identified by LongStrain and found discrete population structures in *B. breve* and *B. longum*. Notably, combining the phylogenetic results with dynamic changes of strains allowed us to directly observe the link between a previously reported subspecies clade of *B. longum* (35) and breastfeeding.

One of LongStrain’s main advantages is its ability to fully utilize repeated measures and reduce noise by preprocessing steps (reads assignment and alignment to representative genomes). Consequently, LongStrain can tackle data with lower sequencing depth compared to other methods like DESMAN and ConStrains. In terms of efficiency, the “Reads Assignment” process of LongStrain demands approximately 8.5 minutes for execution, utilizing around 150 GB of memory, while handling the simulated “Gut20” community (~20 million reads) with a 2.40 GHz CPU (Table 2). Moving to the subsequent phase of “Strain Deconvolution”, LongStrain’s execution duration extends to approximately 139.2 minutes, requiring less than 3 GB of memory. Notably, this timeframe proves to be faster or on par with alternative methods except for ConStrains, as indicated in Table 2.

**TABLE 2:** Computational resources required by five methods for processing the simulated Gut20 community^*a*^

Method	Memory/GB		Time/min	
	Reads assignment	Strain deconvolution	Reads assignment	Strain deconvolution
LongStrain	~150	<3	8.5 (0.2)	139.2 (19.7)
ConStrains	<3		86.9 (20.2)	
MIDAS2	<3		321.4 (22.1)	
StrainPhlAn4	~40		615.0 (36.3)	
DESMAN	–	<3	−	563.6 (87.0)

^*a*^ CPU, Intel(R) Xeon(R) Gold 6148 CPU @ 2.40GHz. “-”, not applicable.

LongStrain exclusively targets the primary strain and potential secondary strains. Consequently, our methodology is particularly well-suited for samples derived from host microbiota, whereas it is not optimal for samples characterized by the presence of multiple strains or frequent strain substitutions, as often encountered in environmental microbiomes. As indicated by our simulation analysis, accurate strain deconvolution with SNV calling in complex strain scenarios (where the number of abundant strains exceeds 2) remains a challenge for LongStrain, as well as for other methods. In such situations, treating the consensus alleles as a dominant strain may be inappropriate. Processing such samples with LongStrain usually yields high estimates (>5%) for the noise strain, indicated by a warning message. Additionally, if the proportions of the primary and secondary strains are consistently close to each other (e.g., 50% to 50%), our method will not correctly assign variants to the strains. Fortunately, this is not a common situation, as samples typically exhibit one dominant strain (20).

Many tools have been developed for the analysis of metagenomic data at strain level, yet each was designed with a specific scope in minds. LongStrain, as a novel pipeline to link genome-wide SNVs with dynamic changes of strain proportions, addresses an important analysis need. In addition, LongStrain’s ability in accurately identifying strain-level SNVs offers a solid foundation for further downstream analyses, such as microbial genome-wide association studies and genomic evolution studies. Given the challenge of deconvolving multiple strains in complex scenarios, there remains a pressing need for further algorithmic enhancements that integrate more accurate databases to advance this field.

## MATERIALS AND METHODS

### Preprocessing of repeated metagenomic sequencing samples

As illustrated in Fig. 1, in LongStrain, the raw metagenomic sequence reads are first assigned to each species by Kraken2 (Fig. 1A). For each species, based on the selected reference genome from the NCBI or GTDB database (Fig. 1B), reads from the same subject are aligned by Bowtie2 and piled up using Pysam within each sample (42). The input to LongStrain’s strain-deconvolution algorithm consists of species-specific read alignments from the repeated metagenomic sequence samples within individual subject (Fig. 1C). To distinguish between strains, LongStrain targets the *effective sites* in the sequence. These are genomic sites where the observed major allele frequency in the aligned reads from all samples is <0.9 or is <0.9 in at least two samples. These filtering criteria are crafted to mitigate noise arising from sequencing and mapping errors.

### Notation preparation

**Fig 5:**
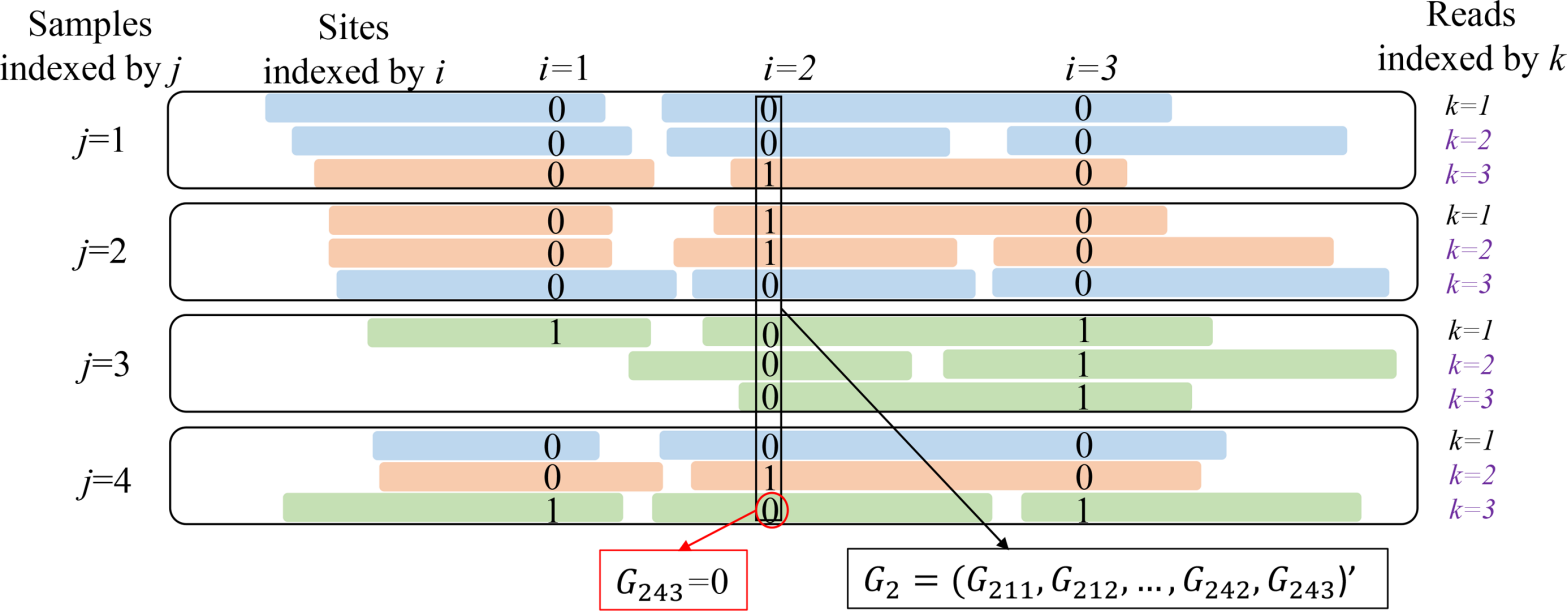
Input data for LongStrain algorithm. For a given species, reads aligned to sites (*i* = 1, 2, 3) from one subject are piled up within four samples (*j* = 1, 2, 3, 4). Strips indicate the reads and different colors indicate different strains. The observed genotype at site *i*, sample *j*, and read *k* is denoted by *G*_ijk_.

As an example, in Fig. 5, for one individual, there are four longitudinal samples. Within each sample, reads (represented by strips) covering the first three consecutive effective sites 1, 2, and 3 on the genome are aligned. Different strip colors indicate different strains. Reads with the same color should share the same genotype at the same effective site (in other words, make up the same haplotypes). As annotated in Fig. 5, we use the letters *i*, *j*, and *k* to index the effective site, sample, and read, respectively. Note that the time order of longitudinal samples is neglected, as LongStrain does not distinguish between longitudinal and parallel samples.

The first step of LongStrain is to use the observed genotype data to classify the reads to one of three different strain types (primary, secondary, or noise). We denote the strain assignment for the *k*^th^ read in the *j*^th^ sample based on the genotype data up to site *i* as *S*_ijk_, and assume that in each sample, there are at most three strains (indexed by *s*, *s* =1, 2, or 3): primary strain, secondary strain, and noise strain. The noise strain is always assumed to be present in the sample such that the undetermined reads and possible errors are absorbed. The strain assignment is achieved by maximizing the likelihood of the genotypes we observed (denoted as *G*_ijk_) under various possible combinations of strain identification. In the second step, with the reads’ strain assignments, we can estimate the proportion of strain s in sample *j* (denoted by *P*_js_) by the ratio of the number of reads classified as strain *s* and the total number of reads in sample *j*, for a certain considered genetic region. We divide the whole genome into windows of 10,000 bp, and each window can contain multiple sites. We update the matrix of proportions for the three hypothesized strains in *J* samples, denoted by


(1) $$P=\left[\begin{array}{ccc} p_{11} & p_{12} & p_{13}\\ p_{21} & p_{22} & p_{23}\\ \vdots & \vdots & \vdots \\ p_{J1} & p_{J2} & p_{J3} \end{array}\right]$$

window by window. For simplicity, we drop the index of windows in all the notations related to *P*. We will introduce how to update *P* in the subsection “Estimation of strain proportions.”

### Algorithm of LongStrain

In order to describe the details of LongStrain algorithm, we divide the effective sites into two categories: (i) non-sharing sites which do not share any reads with prior sites in any of samples (e.g., sites 1 and 2 in Fig. 5). At such sites, only two strains can be differentiated because each site has only two possible genotypes. (ii) Sharing sites, which shares some reads with the prior site (e.g., site 3 in Fig. 5). At such sites, we are able to phase the genotypes according to the haplotypes constructed with prior sites and the strain proportions *P*. Next, we introduce the LongStrain algorithm by site category.

### Category 1: non-sharing sites

Assuming that there are only two strains present, there are only six possible combinations (indexed by *h* in row) of genotypes (0/1) on the three possible strains (indexed by *s* in column) at such site, denoted by a 6 × 3 matrix Θ,


(2) $$\Theta =\left[\begin{array}{ccc} 0 & 1 & NP\\ 1 & 0 & NP\\ NP & 0 & 1\\ NP & 1 & 0\\ 0 & NP & 1\\ 1 & NP & 0 \end{array}\right]$$

where NP indicates that the strain is not present. For example, given *G*_ijk_, the observed genotype of read *k* from sample *j* at site *i*, by matching it to the *h*^th^ row of $\Theta :\Theta_{h}=(\Theta_{h1},\Theta_{h2},\Theta_{h3})`$, we can give the strain identification of this read by:


(3) $${\tilde{S}}_{ijk}^{\left(h\right)}=\sum_{s=1}^{3}I\left[G_{ijk}=\Theta_{hs}\right]s$$

That is, ${\tilde{S}}_{ijk}^{\left(h\right)}$ is the strain index of the only strain compatible with the observed genotype. Then we can calculate the likelihood of observing the genotypes of all reads aligned to site *i*, denoted by $G_{i}=(G_{i11},{\ldots ,G}_{i1n_{1}},\ldots ,G_{iJ1},\ldots ,G_{iJn_{J}})`$ (as illustrated in Fig. 5) with the *h*^th^ combination as


 (4) $$L\left(\Theta_{h}|G_{i}\right)=\prod_{j=1}^{J}\prod_{s=1}^{3}{p_{js}}^{N_{ijs}^{\left(h\right)}}$$

where $N_{ijs}^{\left(h\right)}=\sum_{k=1}^{n_{j}}I\left[G_{ijk}=\Theta_{hs}\right]$ is the number of reads compatible with the given combination of genotypes, *P*_js_ comes from the most updated P matrix in equation 1, and *n*_*j*_ is the number of reads belonging to sample *j* at each site. In practice, *n*_*j*_ varies across sites, but for simplicity of notation, we leave this dependence implicit when describing the method.

We perform the same calculation for *h* = 1, 2, …, 6, and find the combination that maximizes the above likelihood in equation 4, and denote this combination with h*. Then, the strain assignments of reads covering site *i* is obtained by


(5) $${\hat{S}}_{ijk}={\tilde{S}}_{ijk}^{\left(h^{*}\right)}$$

### Category 2: sharing sites

With sharing of reads between the previous and current sites, we are able to differentiate three strains. Since the genotype of each strain can be either 0 or 1, there are totally eight genotype combinations for three strains. Because the *effective sites* that we focus on are biallelic, the genotype combination “0, 0, 0” and “1, 1, 1” are excluded. Thus, there are six possible combinations (indexed by *h* in row) of the genotypes on the three strains (indexed by *s* in column) at such site, denoted by a matrix Θ` with


(6) Θ`=010100001101011110

We define $G_{i}^{shared}$ as the genotype of the reads which also cover site *i* −1 at site *i*, and $G_{i}^{unshared}$as the genotype of the reads that do not cover site *i* −1 at site *i*. We use the vector $U_{i}=(U_{i11},\ldots ,U_{i1n_{1}},\ldots ,U_{ij1},\ldots ,U_{ijn_{j}},\ldots ,U_{iJ1},\ldots ,U_{iJn_{J}})`$ to indicate whether reads covering site *i* also cover site *i* −1 or not. Specifically, if the read at site *i* also covers site *i* −1, *U*_ijk_ = 1; otherwise, *U*_ijk_ = 0.

The likelihood of observing *G*_i_ at site i given the *h*^th^ combination in Θ` can be calculated as


(7) $$L\left(\Theta_{h}^{`}|G_{i}\right)=P\left(G_{i}^{unshared}|\Theta_{h}^{`}\right)P\left(G_{i}^{shared}|\Theta_{h}^{`}\right)$$

For the unshared reads, the probability of observing their genotypes $P\left(G_{i}^{unshared}|\Theta_{h}^{'}\right)$ can be calculated similarly to equation (4) in the first category,


 (8) $$P\left(G_{i}^{unshared}|\Theta_{h}^{`}\right)=\prod_{j=1}^{J}\prod_{s=1}^{3}{p_{js}}^{N_{ijs}^{\left(h\right)}}$$

where $N_{ijs}^{\left(h\right)}=\sum_{k=1}^{n_{j}}I\left[G_{ijk}=\Theta_{hs}^{'}\right]I\left[{\tilde{S}}_{ijk}^{\left(h\right)}=s\right]I\left[U_{ijk}=0\right]$ is the number of unshared reads compatible with the given combination of genotypes and strain index s, and ${\tilde{S}}_{ijk}^{\left(h\right)}=\underset{s}{\mathrm{argmax}}\left\{p_{js}|\Theta_{hs}^{`}=G_{ijk}\right\}$. Note that because there are three possible strains (1, 2, or 3) and only two possible genotype values (0/1), there is chance that *G*_ijk_ matches to two strains. For example, if *G*_ijk_ = 0, for *h* = 1, both strain 1 and strain 3 have genotype 0. In such a case, the definition of ${\tilde{S}}_{ijk}^{\left(h\right)}$ and indicator function $I\left[{\tilde{S}}_{ijk}^{\left(h\right)}=s\right]$ ensure that the read is assigned to the strain whose *P*_js_ is larger.

$P\left(G_{i}^{shared}|\Theta_{h}^{`}\right)$ is the probability of observing genotypes of the shared reads. Since the shared reads have already been assigned to a strain at the previous site *P*_js_, we denote the strain identity estimated from the previous site for the *k*^th^ read in the *j*^th^ sample at *i*^th^ site as *S*_ijk_. For notational simplicity, when *U*_ijk_ = 0, we will arbitrarily set *S*_ijk_ = 1 to avoid the undefined value in the following equation 9.


(9) $$P\left(G_{i}^{shared}|\Theta_{h}^{'}\right)=\prod_{j=1}^{J}\sum_{k=1}^{n_{j}}{\left({\left(1-\varepsilon \right)}^{I\left[G_{ijk}=\Theta_{h{\overset{ˇ}{S}}_{ijk}}^{'}\right]}{f_{jk}}^{I\left[G_{ijk}\neq \Theta_{h{\overset{ˇ}{S}}_{ijk}}^{'}\right]}\right)}^{U_{ijk}}$$

where $f_{jk}=\left\{ \begin{array}{cl} \varepsilon & {\bar{\bar{S}}}_{ijk}^{\left(h\right)}=\emptyset \\ \frac{p_{j{\bar{s}}_{ijk}^{\left(h\right)}}}{p_{j{\bar{\bar{s}}}_{ijk}^{\left(h\right)}}+p_{j{\bar{Š}}_{ijk}}} & {\bar{\bar{S}}}_{ijk}^{\left(h\right)}\neq \emptyset^{b}{\bar{\bar{S}}}_{ijk}^{\left(h\right)}=\left\{s|\Theta_{hs}^{\prime }=G_{ijk}\right\}\cap {\tilde{S}}_{\left(i-1\right)} \end{array}\right.$

In equation 9, ε is the error caused by sequencing or mapping and is set to 0.001 (55) for simplicity of computation. When $G_{ijk}=\Theta_{h{Š}_{ijk}}^{\prime }$, the genotype of read *k* in sample *j* is consistent with the strain identity obtained at the previous site, we keep its strain identity and assign the probability to observe *G*_ijk_ as 1 − ε. When $G_{ijk}\neq \Theta_{h{\overset{ˇ}{S}}_{ijk}}^{`}$, the observed genotype is inconsistent with the inferred strain from the previous site, we need to decide which strain the read belongs to and what is the probability of observing the genotype. Let${\tilde{s}}_{\left(i-1\right)}$ denote the strain that is not detected at site *i* −1. For example, if strain 1 and strain 3 are detected at the previous site, then ${\tilde{s}}_{\left(i-1\right)}=\left\{2\right\}.$ If ${\bar{\bar{S}}}_{ijk}^{\left(h\right)}\neq \emptyset$, there is one strain that is not detected at the previous site and whose genotype agrees with *G*_ijk_. In other words, read *k* which is assigned to *S*_ijk_ at site *i* −1 is more likely from ${\bar{\bar{S}}}_{ijk}^{\left(h\right)}$. If ${\bar{\bar{S}}}_{ijk}^{\left(h\right)}=\emptyset$, the inconsistency between *G*_ijk_ and $\Theta_{h{\overset{ˇ}{S}}_{ijk}}^{`}$ will be attributed to the sequencing or mapping error. We perform the calculation for *h* = 1, 2, …, 6 to find the *h* that maximizes the likelihood in equation 7 and denote it as h*. Then, the strain identification *S*_ijk_ can be obtained according to the following rules:


(10) $${Š}_{ijk}=\left\{ \begin{array}{l} {\tilde{S}}_{ijk}^{\left(h^{*}\right)},\;\text{ if the }i^{th}\text{ read is unshared }\\ {\overset{ˇ}{S}}_{ijk},\text{ if the }i^{th}\text{ read is shared and }G_{ijk}=\Theta_{h*{\overset{ˇ}{S}}_{ijk}}^{\prime }\\ {\overset{ˇ}{S}}_{ijk}\text{, if the }i^{\text{th }}\text{ read is shared and }G_{ijk}\neq \Theta_{h*{\overset{ˇ}{S}}_{ijk}}^{\prime }\text{ and }{\bar{\bar{S}}}_{ijk}^{\left(h*\right)}=\emptyset \\ {\bar{\bar{S}}}_{ijk}^{\left(h*\right)},\text{ if the }i^{th}\text{ read is shared and }G_{ijk}\neq \Theta_{h*{\overset{ˇ}{s}}_{ijk}}^{\prime }\text{ and }{\bar{\bar{S}}}_{ijk}^{\left(h*\right)}\neq \emptyset \end{array}\right.$$

### Estimation of strain proportions

Finally, we introduce how strain-proportion matrix *P* is initialized and updated. We define *C* as the count matrix of reads from three hypothesized strains in *J* samples.


$$C=\left[\begin{array}{ccc} c_{11} & c_{12} & c_{13}\\ c_{21} & c_{22} & c_{23}\\ \vdots & \vdots & \vdots \\ c_{J1} & c_{J2} & c_{J3} \end{array}\right],$$

where *C*_js_ represents the cumulative count of reads from strain *s* in sample *j*. *P*_js_ is calculated as


$$p_{js}=\frac{c_{js}}{\sum_{s^{\prime }=1}^{3}c_{js^{\prime }}}.$$

We divided the whole genome into windows of 10,000 bp and calculate the average depth of each window. After sorting all the windows by their average depth, we selected five windows as close as possible to the 80th quantile. We initialized the analysis in these five windows with an equal starting value of $p_{is}=\frac{1}{3}$. Using the algorithm introduced above, we assigned reads in these regions to three hypothesized strains and obtain the initial *P* and *C*. Then, we screened the whole genome and updated *P* and *C* window by window until all are finished.

### Simulation data analysis

To compare LongStrain with four competing methods in a more realistic situation, we simulated a gut microbial community (Gut20) which consisted of 20 species used in the previous studies (43, 44). Since *Actinomyces odontolyticus*, *Bacteroides vulgatus*, *Propionibacterium acnes*, *Streptococcus pneumoniae*, and *Deinococcus radiodurans* did not have enough complete genomes of high quality in the GTDB database, we replaced them with five other common species in human gut (*B. breve*, *B. adolescentis*, *B. fragilis*, *B. longum*, and *B. bifidum*). For each species, we chose two complete genomes at random from the GTDB database. Detailed information about these genomes, including genome ID, qualities, and ANIs, is provided in Table S11. To mimic longitudinal changes in the abundance of two strains, we conceived the following scenarios for sequencing depths of two strains at three time points: [9:1, 9:1, 9:1], [8:2, 8:2, 8:2], [7:3, 7:3, 7:3], [6:4, 6:4, 6:4], [9:1, 8:2, 7:3], [9:1, 7:3, 5:5], [9:1, 6:4, 3:7], [9:1, 5:5, 1:9], [8:2, 6:4, 4:6], [8:2, 7:3, 3:7]. For example, [9:1, 9:1, 9:1] represents average sequencing depths of 9× and 1× for each of the two strains, respectively, at all three time points. In each repetition, every species was simulated according to a scenario randomly selected from the pre-defined scenarios. Paired HiSeq-2000 reads with the length of 100 bp were generated from these two genomes using ART 2.5.8 with command “art_illumina -ss HS20 -p -l 100 m 200 s 10.” At each time point, simulated reads of all 20 species were pooled together to get the microbial community data.

In the simulation analysis, the performance of LongStrain was compared with four methods, ConStrains, MIDAS2, StrainPhlAn4, and DESMAN 2.1. Since strain deconvolution is not the goal of StrainPhlAn4 and MIDAS2 and they do not output such results, just for the purpose of comparison, we treated the consensus alleles identified by these two methods as their dominant strains, and compared them to the primary strains reported by DESMAN and LongStrain. The performances of these methods were evaluated by the recall and accuracy of the identified SNVs, with the exception of ConStrains. The estimation of strain proportions was assessed across LongStrain, DESMAN, and ConStrains. As DESMAN and ConStrains do not furnish the GTDB database, the GTDB database was employed in the analyses by LongStrain and MIDAS2. For MIDAS2, the raw sequencing data were processed by the command “run_species --midasdb_name gtdb” at each time point and merged as a final result using the command “merge_species --min_cov 2.” The variants across the samples were merged using the commands “run_snps --select_by median_marker_coverage,unique_fraction_covered --select_threshold = 2,0.5” and “merge_snps --site_depth 1 --site_ratio 10 --snp_maf 0.001 --site_prev 0.01 --snp_pooled_method abundance.” All sites with a dominant allele that was different from the reference genome were regarded as variants of the primary strain. For StrainPhlAn4, consensus sequences were obtained at each time point by “sample2markers.py” and “extract_markers.py” with default parameters. Subsequently, the phylogenetic trees were constructed under “accurate” mode of “strainphlan” with default parameters. As with MIDAS2, SNVs identified by StrainPhlAn4 were also defined as genotypes different from the reference genome. However, StrainPhlAn4 cannot merge results from different time points, and we therefore calculated the accuracies of StrainPhlAn at each time point separately. The strain proportions, as estimated by ConStrains, were acquired utilizing default parameters. LongStrain, in turn, involved the alignment of reads classified by Kraken2 to representative genomes from the GTDB using Bowtie2, employing default parameters. Following this alignment, the resultant reads underwent a pile-up procedure conducted by Pysam, with specified parameters “min_base_quality = 13, min_mapping_quality = 20.” The output of this process served as input to LongStrain’s strain-deconvolution algorithm. Furthermore, the input to LongStrain’s strain-deconvolution algorithm underwent the strain-deconvolution processing via DESMAN with specified parameters “desman -g 2 -i 50” (switching to “-g 3” for three-strain scenarios).

To calculate the accuracy and recall of the identified SNVs, we had to get the true SNVs of the strains relative to the reference genome. Therefore, we generated reads from the original genomes of the strains to a high depth of 100× using ART (56). These reads were mapped to the reference genomes in MIDAS database by Bowtie2 (40) with default parameters. The horizontal genome coverage for each species is summarized in Table S12. Then, SNVs were called by SAMtools 1.9 (42) with the command “samtools mpileup -uf reference.fas sample.bam | bcftools call -c --output-type v -v.” These SNVs were regarded as the gold standard in the calculation of recall and accuracy. The proportion of SNVs identified by each method overlapping with the gold standard was calculated as the precision. The recall of each method was the proportion of variants in the gold standard that were correctly detected by that method.

For the comparison with ConStrains and DESMAN, the MAE and RMSE of proportion estimate were calculated as follows:

MSE = $\frac{1}{N}\sum_{i=1}^{N}\frac{1}{3}\sum_{j=1}^{3}\left|p_{i,j}^{primary}{\hat{p}}_{i,j}^{primary}\right|$; RMSE = $\sqrt{\frac{1}{N}\sum_{i=1}^{N}\frac{1}{3}\sum_{j=1}^{3}{\left(p_{i,j}^{primary}-{\hat{p}}_{i,j}^{primary}\right)}^{2}}$ , where *N* is the number of successful reports in 20 repetitions of the simulation, *i* is the index of repeat, *j* is the index of time point, and *P^primary^* is the proportion of the primary strain.

### Real data analysis

#### Analysis of a subset of TEDDY

In this study, we only focused on the subjects with T1D case-control status which consisted of 100 cases and 100 controls. We used LongStrain to screen 44 high-abundance species mentioned in two published TEDDY microbiome studies (34, 45).

### Association between strain proportions/diversity and birth delivery mode

The log2-ratio of the proportions of the primary strain to the secondary strain at each time point was calculated by $\log_{2}\left(\frac{primary\;strain\;proportion}{secondary\;strain\;proportion}\right)$. To reduce the noise, given each species, only time points with >3,000 reads were retained and the log2-ratios were averaged over a sliding window of 2 months. To ensure an adequate sample size and include strain transitions, we focused on 26 species and used the samples from the first 2 years. We fitted the following linear mixed effect model to test whether the temporal trend of the log2-ratio was associated with the birth delivery mode.


$$\log_{2}Ratio\sim \beta_{0}+\beta_{1}Month+\beta_{2}BirthMode+\beta_{3}Month\times BirthMode+\beta_{4}Sex+\left(1|subject\_ID\right),$$

where “*BirthMode*” is the birth delivery mode (cesarean or vaginal) and “*subject_ID*” is a random effect. The sex was adjusted in this model and *P*-values were reported after FDR correction. Shannon’s index of strain proportion is calculated by $H=-\sum_{i=1}^{3}p_{i}\ln p_{i}$, where $p_{i}$ is the proportion of *i*^th^ strain at a given time point. Similarly, we fitted the linear mixed effect model below to test the association between the temporal trend of strain diversity and the birth delivery mode.


$$H_{shannon}\sim \beta_{0}+\beta_{1}Month+\beta_{2}BirthMode+\beta_{3}Month\times BirthMode+\beta_{4}Sex+\left(1|subject\_ID\right).$$

## Data Availability

Data from the TEDDY study (DOI: 10.58020/y3jk-x087) reported here are available for request via the NIDDK Central Repository, Resources for Research (R4R) at https://repository.niddk.nih.gov with accession number phs001442.v4.p3. For the gastric intestinal metaplasia microbiome study, the metagenomic sequencing data are available in the dbGaP at https://www.ncbi.nlm.nih.gov/gap/ with accession number phs002566.v1.p1. LongStrain is implemented in Python 3 on Linux and is freely available, along with step-by-step tutorials, at https://github.com/BoyanZhou/LongStrain under the Apache-2.0 license.

## SUPPLEMENTAL MATERIAL

Supplemental figures and tables spectrum.01431-24-s0001.docx Fig. S1 to S8; Tables S1 to S12.

ASM does not own the copyrights to Supplemental Material that may be linked to, or accessed through, an article. The authors have granted ASM a non-exclusive, world-wide license to publish the Supplemental Material files. Please contact the corresponding author directly for reuse.
