# Supplementary material for: An integrated strain-level analytic pipeline utilizing longitudinal metagenomic data
Source: Microbiol Spectr. 2024 Sep 23;12(11):e01431-24. doi: 10.1128/spectrum.01431-24 (PMC11542597; doi:10.1128/spectrum.01431-24)
Supplement: Supplemental figures and tables — Fig. S1 to S8; Tables S1 to S12. [file spectrum.01431-24-s0001.docx]

**An integrated strain-level analytic pipeline utilizing longitudinal metagenomic data**

Boyan Zhou^1^, Chan Wang^1^, Gregory Putzel^2^, Jiyuan Hu^1^, Menghan Liu^3^, Fen Wu^4^, Yu Chen^4^, Alejandro Pironti^2^, Huilin Li^1#^

1 Division of Biostatistics, Department of Population Health, New York University School of Medicine, New York, New York 10016, USA

2 Department of Microbiology, New York University School of Medicine, New York, New York 10016, USA

3 Department of Biological Sciences, Columbia University in the City of New York, New York, New York 10027, USA

4 Division of Epidemiology, Department of Population Health, New York University School of Medicine, New York, New York 10016, USA

Correspondence: [Huilin.Li@nyulangone.org](mailto:Huilin.Li@nyulangone.org)

**Supplemental Material**

Contents

[The full list of 44 screened species in the real data analysis of TEDDY dataset 4](#_Toc165375983)

[Supplemental Figures 5](#_Toc165375984)

[Supplemental Figure S1. Comparison of LongStrain, MIDAS2, DESMAN, and StrainPhlan4 in single species (*Bifidobacterium breve*) simulation across 20 repetitions at the sequencing depth of 10$\times$. 5](#_Toc165375985)

[Supplemental Figure S2. Number of samples that have a strain with *p*>80% for 26 species in the TEDDY dataset by LongStrain. 6](#_Toc165375986)

[Supplemental Figure S3. Number of subjects with/without a strain transition for 26 species in the TEDDY dataset by LongStrain. A “strain transition” is defined as the occurrence wherein a dominant strain (*p*>=50%) shifts to a non-dominant status (*p*<50%) within longitudinal samples of a subject. 7](#_Toc165375987)

[Supplemental Figure S4. The scatter plots of time of the first transition and early life events (antibiotic treatment and weaning) in the TEDDY dataset. 8](#_Toc165375988)

[Supplemental Figure S5. Distribution of strain similarities in *Akkermansia muciniphila* in the TEDDY dataset. 9](#_Toc165375989)

[Supplemental Figure S6. Distribution of strain similarities in *Bacteroides uniformis* in the TEDDY dataset. 10](#_Toc165375990)

[Supplemental Figure S7. Distribution of strain similarities in *Bifidobacterium longum* in the TEDDY dataset. 11](#_Toc165375991)

[Supplemental Figure S8. Phylogenetic tree of six species in the TEDDY dataset: 12](#_Toc165375992)

[Supplemental Tables 13](#_Toc165375993)

[Supplemental Table S1. Comparison of LongStrain, MIDAS2, DESMAN, and StrainPhlan4 in precision of SNV calling in community simulation (Gut20) across 20 repetitions 13](#_Toc165375994)

[Supplemental Table S2. Comparison of LongStrain, MIDAS2, DESMAN, and StrainPhlan4 in recall of SNV calling in community simulation (Gut20) across 20 repetitions 14](#_Toc165375995)

[Supplemental Table S3. Comparison of LongStrain, MIDAS2, DESMAN, and StrainPhlan4 in precision of SNV calling in single-species simulation (*Bifidobacterium breve*) in two scenarios at different depths across 20 repetitions 15](#_Toc165375996)

[Supplemental Table S4. Comparison of LongStrain, MIDAS2, DESMAN, and StrainPhlan4 in recall of SNV calling in single-species simulation (*Bifidobacterium breve*) in two scenarios at different depths across 20 repetitions 15](#_Toc165375997)

[Supplemental Table S5. The accuracy of the estimated proportion of the primary strain by ConStrains, DESMAN, and LongStrain in single-species simulation (*Bifidobacterium breve*) in two scenarios at different depths across 20 repetitions 16](#_Toc165375998)

[Supplemental Table S6. Comparison of LongStrain, MIDAS2, DESMAN, and StrainPhlan4 in precision of SNV calling in single-species simulation (*Bifidobacterium breve*) in two scenarios using references with different ANIs (relative to the representative genome) at sequencing depth of 10$\times$ across 20 repetitions 16](#_Toc165375999)

[Supplemental Table S7. Comparison of LongStrain, MIDAS2, DESMAN, and StrainPhlan4 in recall of SNV calling in single-species simulation (*Bifidobacterium breve*) in two scenarios using references with different ANIs (relative to the representative genome) at sequencing depth of 10$\times$ across 20 repetitions 17](#_Toc165376000)

[Supplemental Table S8. The accuracy of the estimated proportion of the primary strain by ConStrains, DESMAN, and LongStrain in single-species simulation (*Bifidobacterium breve*) in two scenarios using references with different ANIs (relative to the representative genome) at sequencing depth of 10$\times$ across 20 repetitions 17](#_Toc165376001)

[Supplemental Table S9. The accuracy of the estimated proportion of the primary strain by ConStrains, DESMAN, and LongStrain in single-species simulation (*Bifidobacterium breve*) in three-strain scenario at depth of 10$\times$ 18](#_Toc165376002)

[Supplemental Table S10. Association test of the log2-ratio (primary strain proportion/secondary strain proportion) and the birth mode (caesarian or vaginal) by the linear mixed model 19](#_Toc165376003)

[Supplemental Table S11. Detailed information of strain genomes used in community simulation 20](#_Toc165376004)

[Supplemental Table S12. Horizontal genome coverage for each species in community simulation 21](#_Toc165376005)

# The full list of 44 screened species in the real data analysis of TEDDY dataset

*Bifidobacterium bifidum*, *Bifidobacterium breve*, *Bifidobacterium catenulatum*, *Bifidobacterium longum*, *Bifidobacterium pseudocatenulatum*, *Bifidobacterium dentium*, *Bifidobacterium adolescentis*, *Bacteroides caccae*, *Bacteroides fragilis*, *Bacteroides sp. A1C1*, *Bacteroides thetaiotaomicron*, *Bacteroides uniformis*, *Bacteroides vulgatus*, *Staphylococcus aureus*, *Streptococcus mitis*, *Streptococcus oralis*, *Streptococcus parasanguinis*, *Streptococcus pneumoniae*,

*Streptococcus pseudopneumoniae*, *Streptococcus sp. 116-D4*, *Streptococcus thermophilus*, *Staphylococcus epidermidis*, *Lactobacillus rhamnosus*, *Lactococcus lactis*, *Enterococcus avium*, *Enterococcus faecalis*, *Enterococcus faecium*, *Enterococcus casseliflavus*, *Eggerthella lenta*, *Roseburia hominis*, *Rothia mucilaginosa*, *Clostridium butyricum*, *Clostridium perfringens*, *Anaerostipes hadrus*, *Anaerostipes rhamnosivorans*, *Roseburia intestinalis*, *Lachnospiraceae bacterium GAM79*, *Faecalibacterium prausnitzii*, *Flavonifractor plautii*, *Ruthenibacterium lactatiformans*, *Veillonella parvula*, *Escherichia coli*, *Akkermansia muciniphila*, *Helicobacter pylori*.

# Supplemental Figures

**
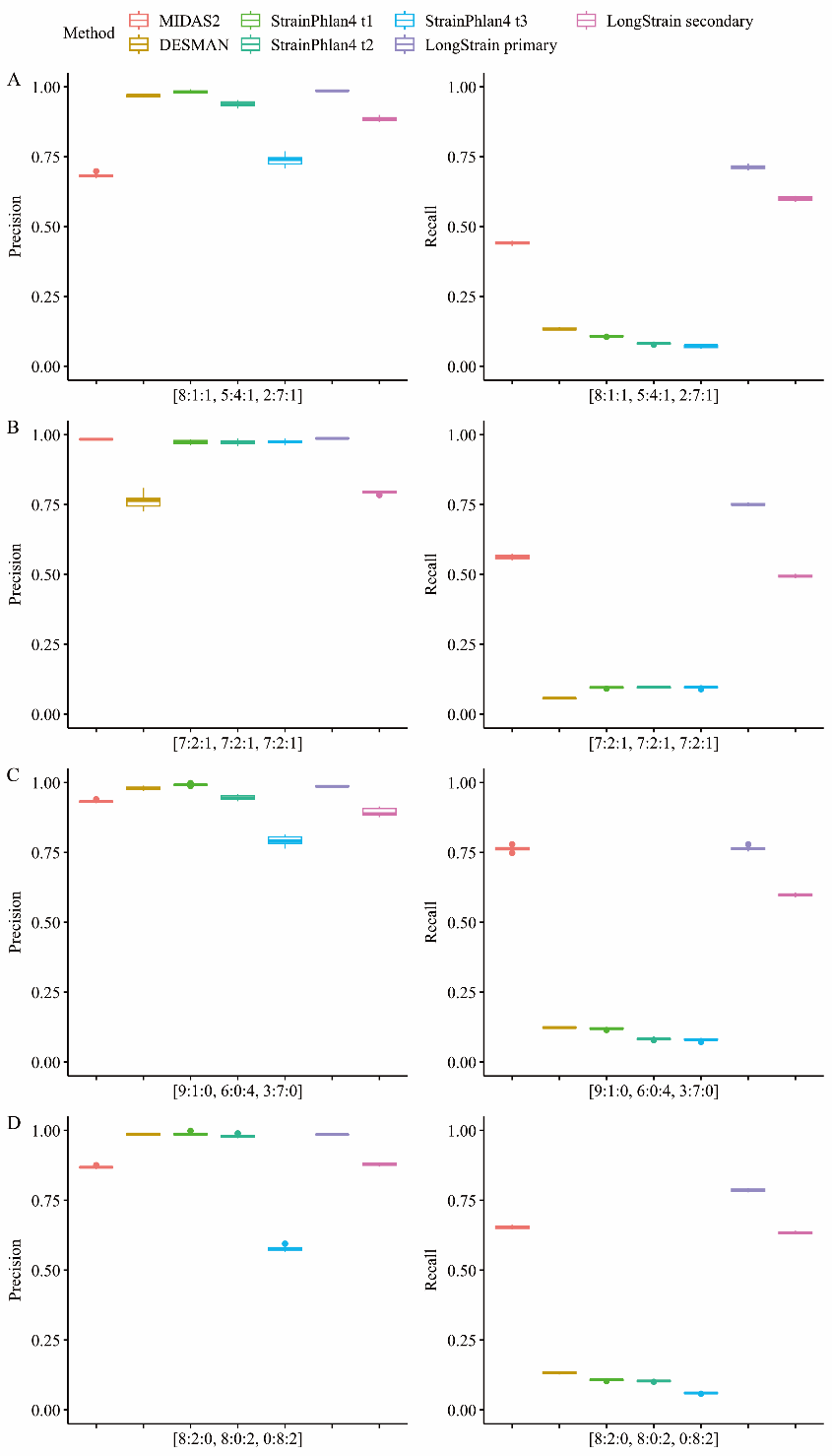
**

Supplemental Figure S1. Comparison of LongStrain, MIDAS2, DESMAN, and StrainPhlan4 in single species (*Bifidobacterium breve*) simulation across 20 repetitions at the sequencing depth of 10$\times$. **A** Precision and recall in scenario [8:1:1, 5:4:1, 2:7:1]; **B** Precision and recall in scenario [7:2:1, 7:2:1, 7:2:1]; **C** Precision and recall in scenario [9:1:0, 6:0:4, 3:7:0]; **D** Precision and recall in scenario [8:2:0, 8:0:2, 0:8:2].

**
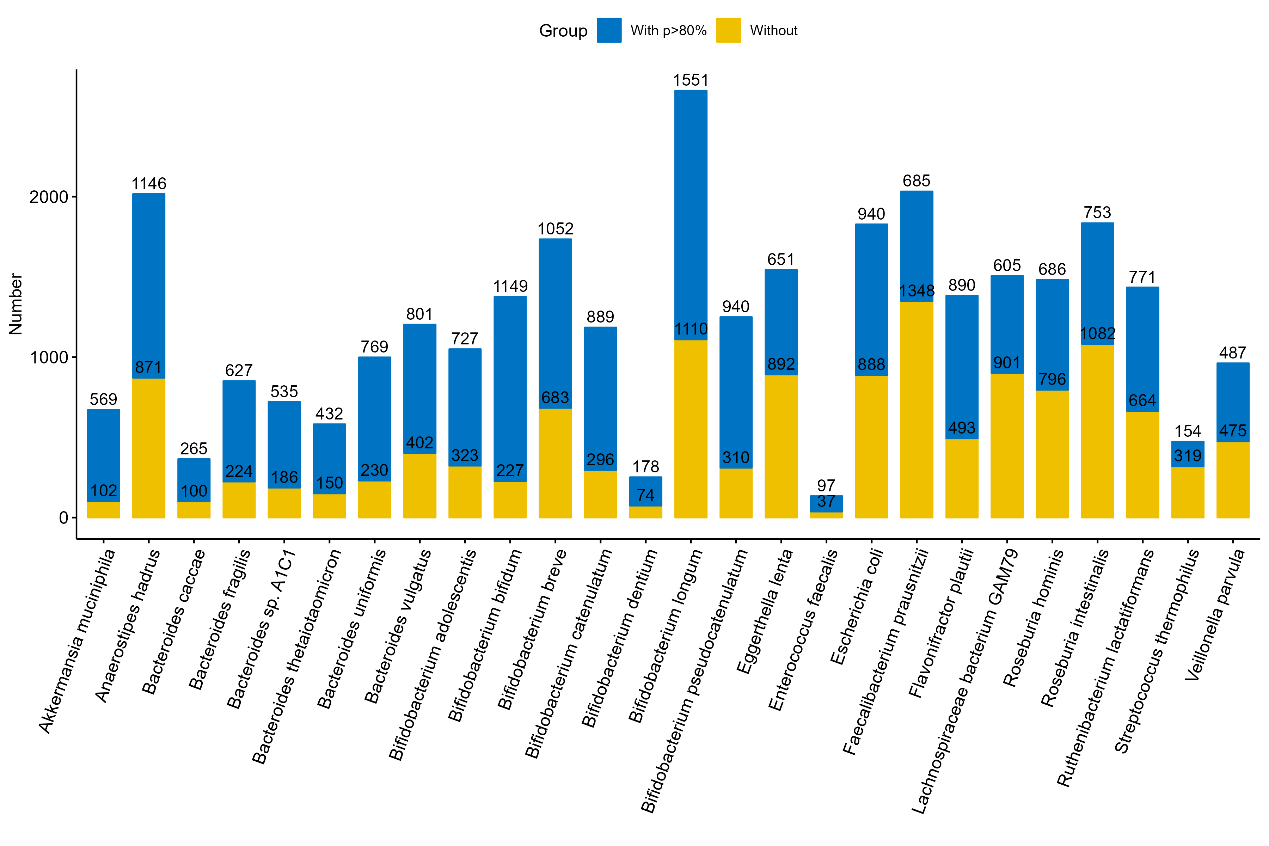
**

Supplemental Figure S2. Number of samples that have a strain with *p*>80% for 26 species in the TEDDY dataset by LongStrain. *p* is defined as the proportion of a strain within its species at single time points. Blue: samples with a strain *p*>80% for a given species; yellow: samples without a strain *p*>80% for a given species**.**


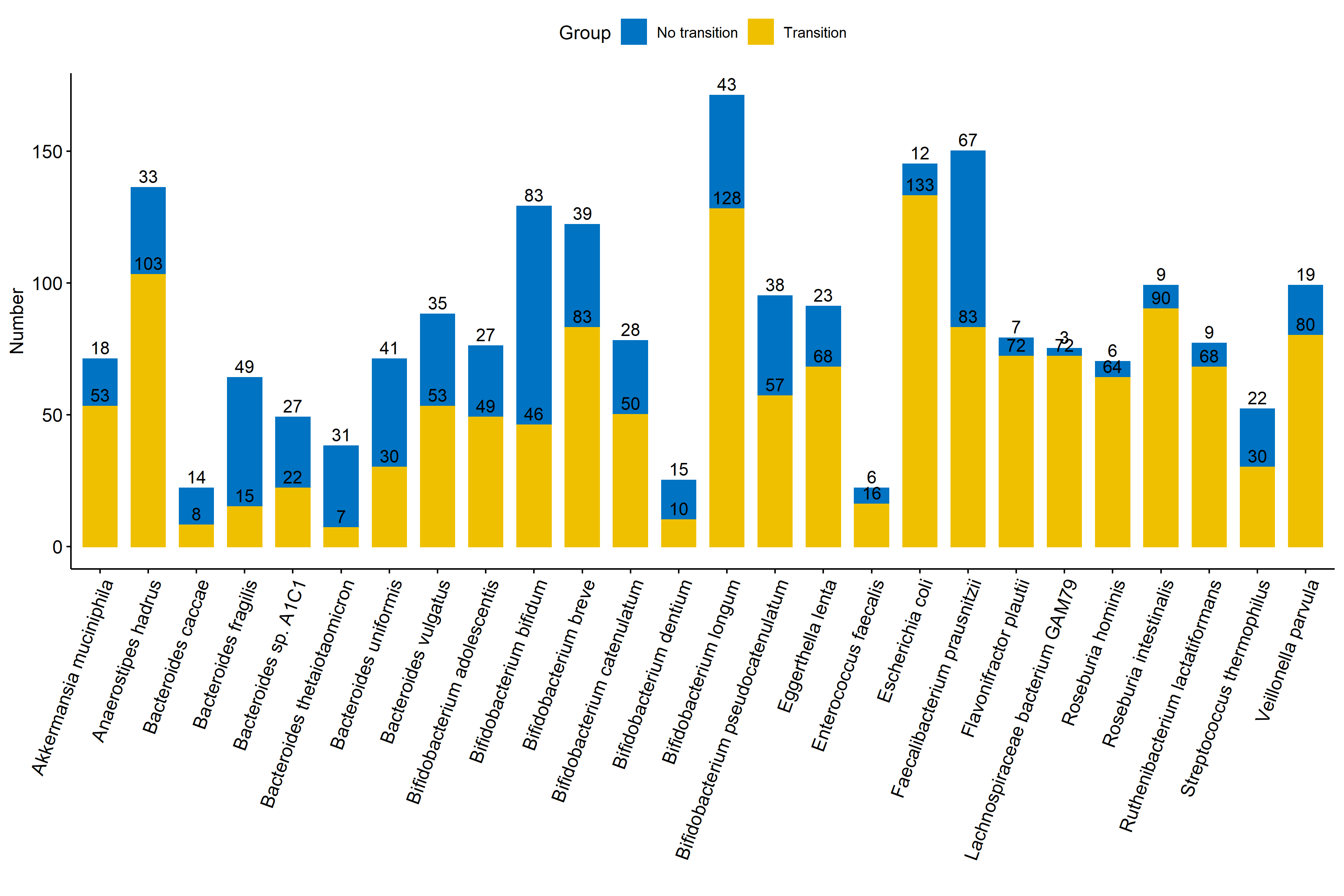


Supplemental Figure S3. Number of subjects with/without a strain transition for 26 species in the TEDDY dataset by LongStrain. A “strain transition” is defined as the occurrence wherein a dominant strain (*p*>=50%) shifts to a non-dominant status (*p*<50%) within longitudinal samples of a subject. We summarized whether a strain transition happened in all subjects with sufficient read counts. Blue: subjects without a strain transition; yellow: subjects with at least one strain transition.


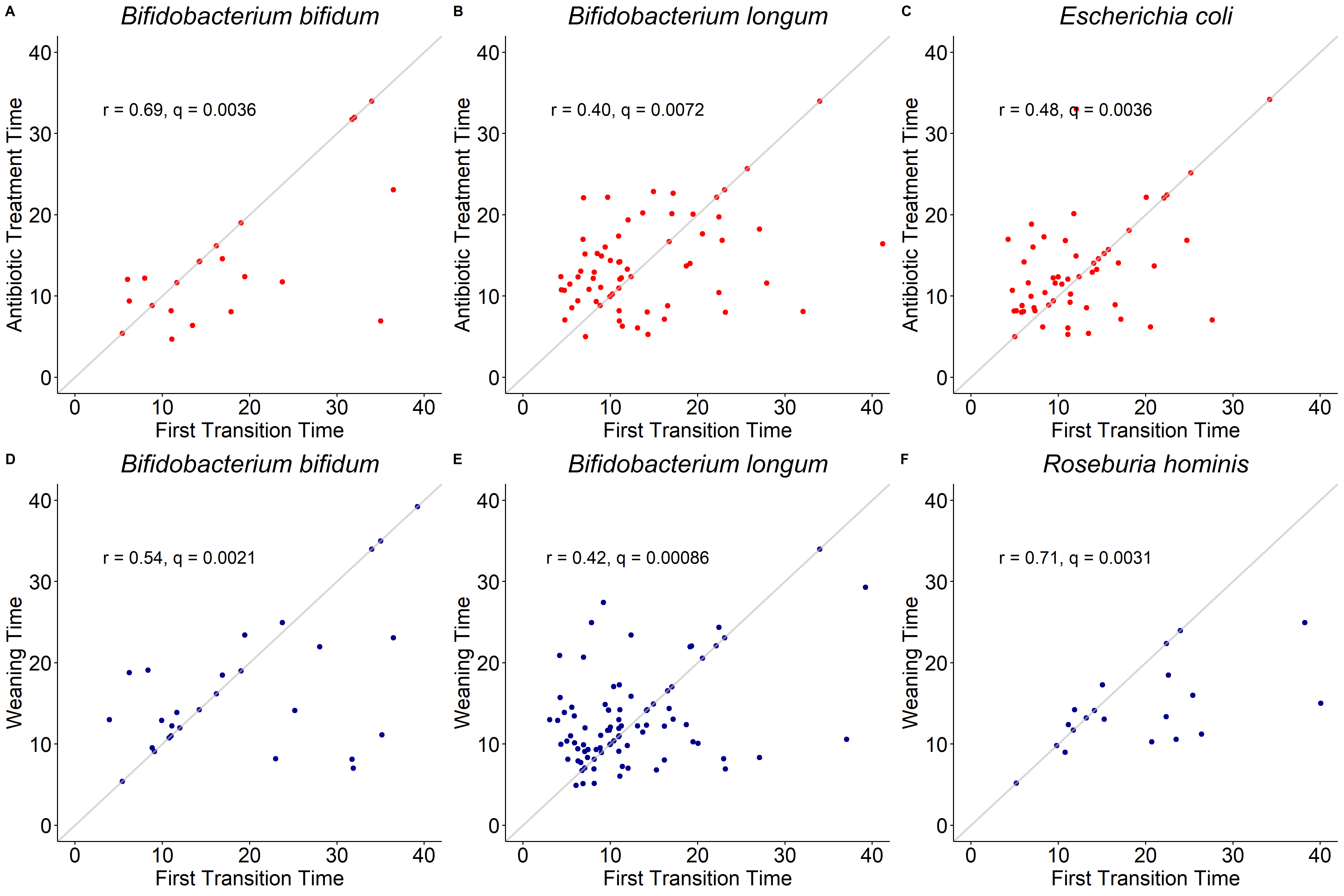


Supplemental Figure S4. The scatter plots of time of the first transition and early life events (antibiotic treatment and weaning) in the TEDDY dataset. A. Scatter plot and Pearson correlation test between the time of the first transition and antibiotic treatment in *Bifidobacterium bifidum*; B. scatter plot and Pearson correlation test between the time of the first transition and antibiotic treatment in *Bifidobacterium longum*; C. scatter plot and Pearson correlation test between the time of the first transition and antibiotic treatment in *Escherichia coli*; D. scatter plot and Pearson correlation test between the time of the first transition and weaning in *Bifidobacterium bifidum*; E. scatter plot and Pearson correlation test between the time of the first transition and weaning in *Bifidobacterium longum*; F. scatter plot and Pearson correlation test between the time of the first transition and weaning in *Roseburia hominis*.


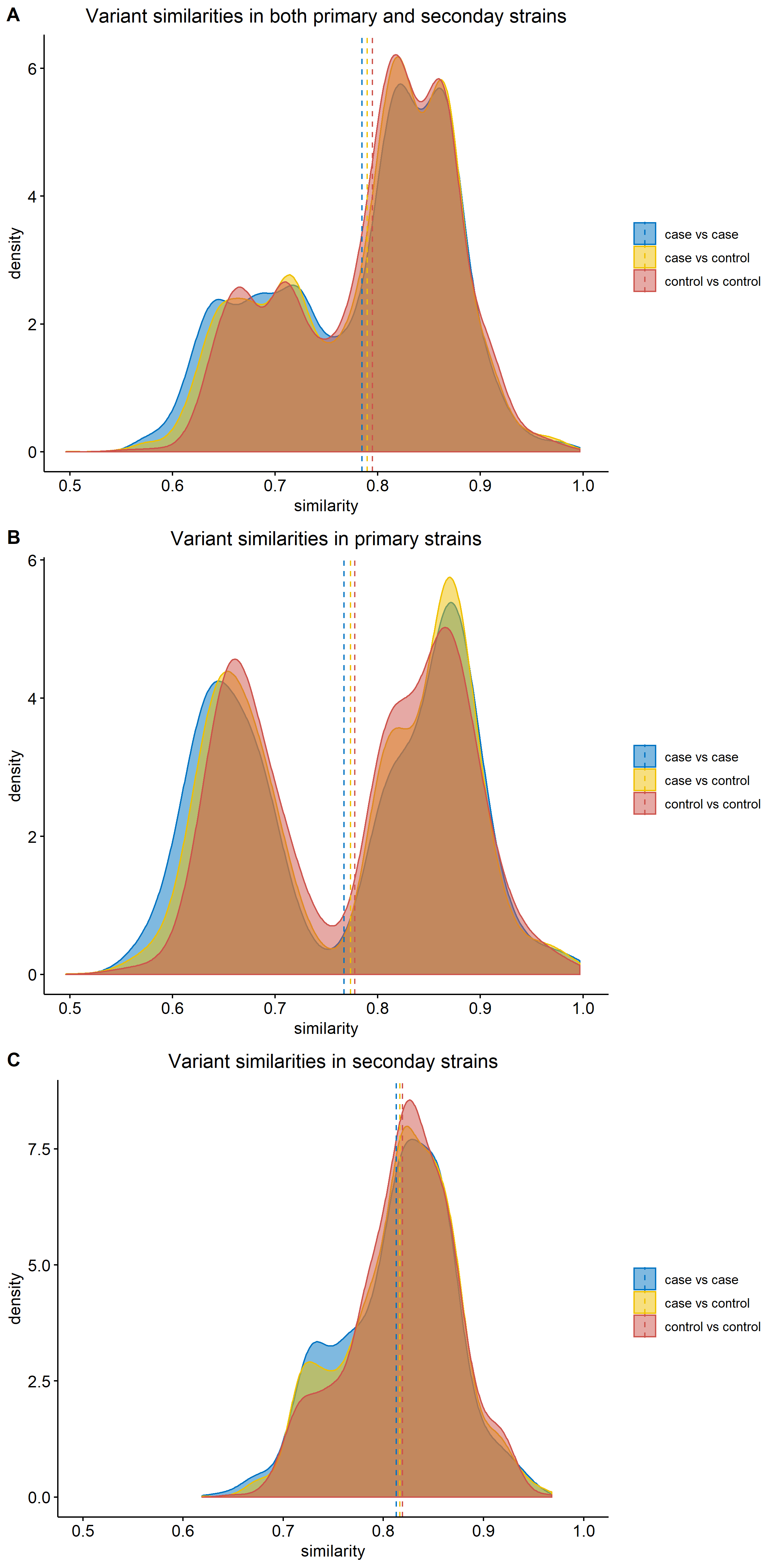


Supplemental Figure S5. Distribution of strain similarities in *Akkermansia muciniphila* in the TEDDY dataset. **A** Strain similarities of “*case vs case*”, “*case vs control*”, and “*control vs control*” in both primary and secondary strains; **B** strain similarities of “*case vs case*”, “*case vs control*”, and “*control vs control*” in primary strains; **C** strain similarities of “*case vs case*”, “*case vs control*”, and “*control vs control*” in secondary strains.


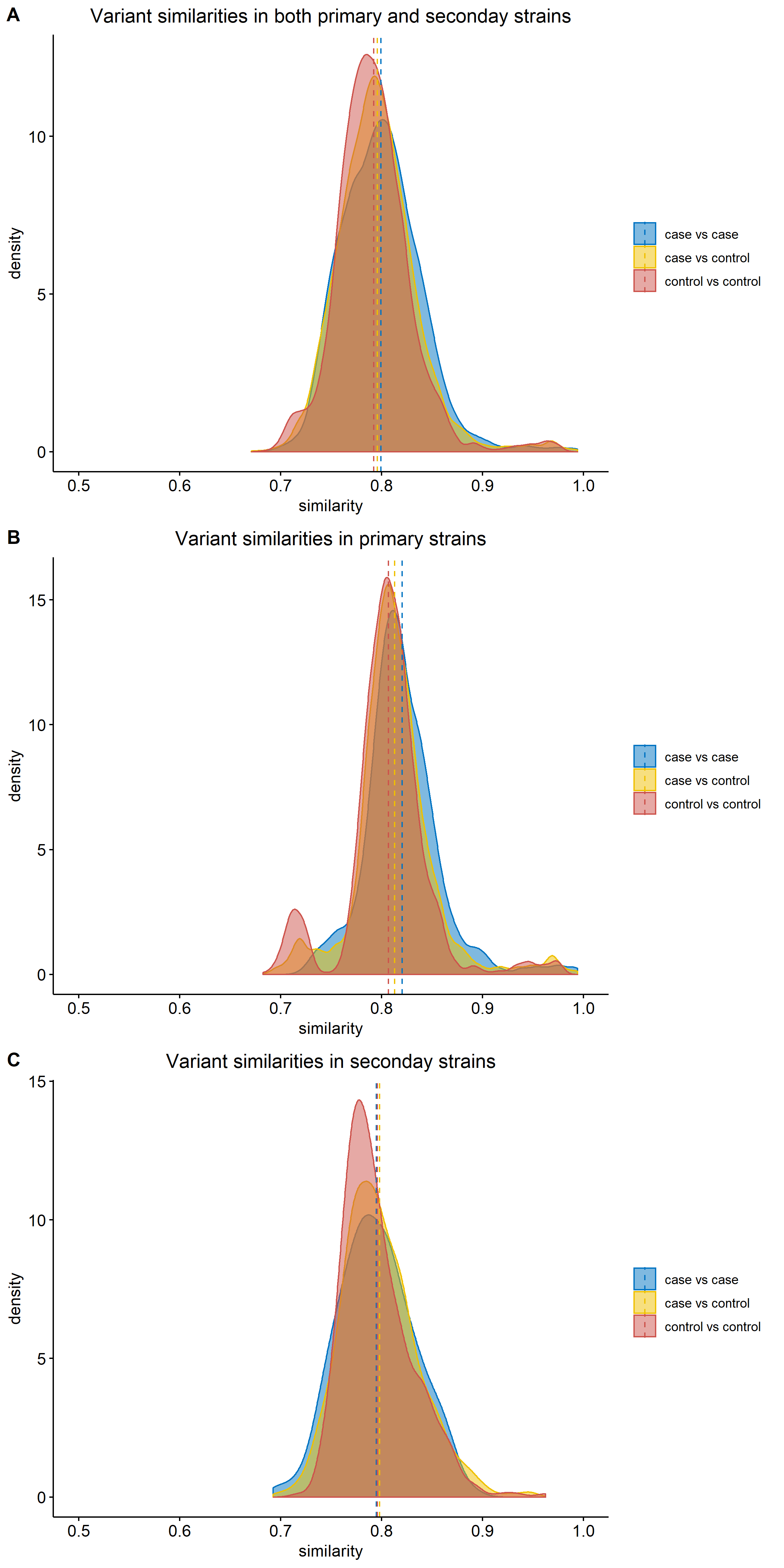


Supplemental Figure S6. Distribution of strain similarities in *Bacteroides uniformis* in the TEDDY dataset. **A** Strain similarities of “*case vs case*”, “*case vs control*”, and “*control vs control*” in both primary and secondary strains; **B** strain similarities of “*case vs case*”, “*case vs control*”, and “*control vs control*” in primary strains; **C** strain similarities of “*case vs case*”, “*case vs control*”, and “*control vs control*” in secondary strains.


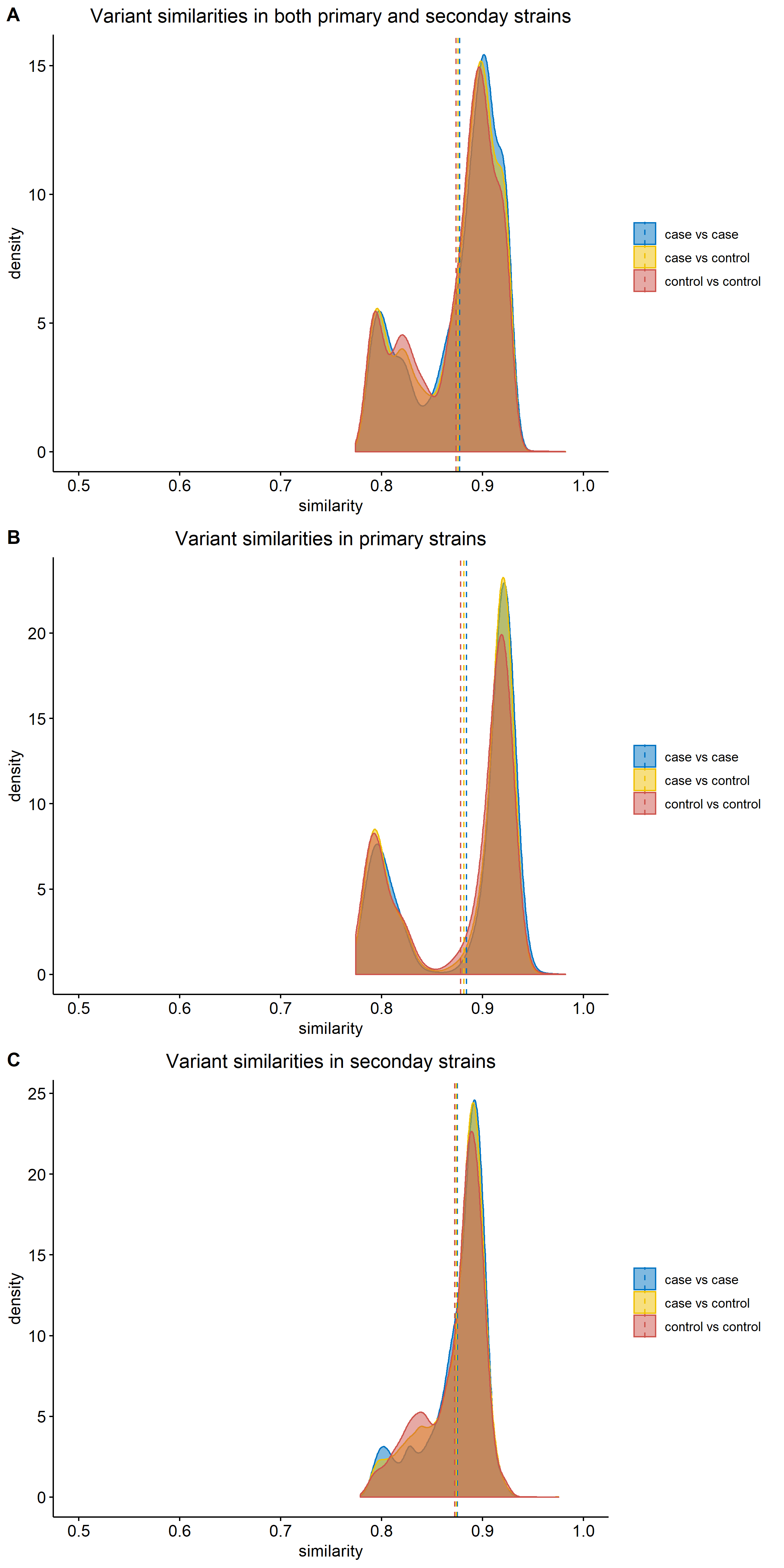


Supplemental Figure S7. Distribution of strain similarities in *Bifidobacterium longum* in the TEDDY dataset. **A** Strain similarities of “*case vs case*”, “*case vs control*”, and “*control vs control*” in both primary and secondary strains; **B** strain similarities of “*case vs case*”, “*case vs control*”, and “*control vs control*” in primary strains; **C** strain similarities of “*case vs case*”, “*case vs control*”, and “*control vs control*” in secondary strains.

Supplemental Figure S8. Phylogenetic tree of six species in the TEDDY dataset: *Akkermansia muciniphila*, *Bifidobacterium bifidum*, *Bacteroides uniformis*, *Bacteroides vulgatus*, *Eggerthella lenta*, *Faecalibacterium prausnitzii*.


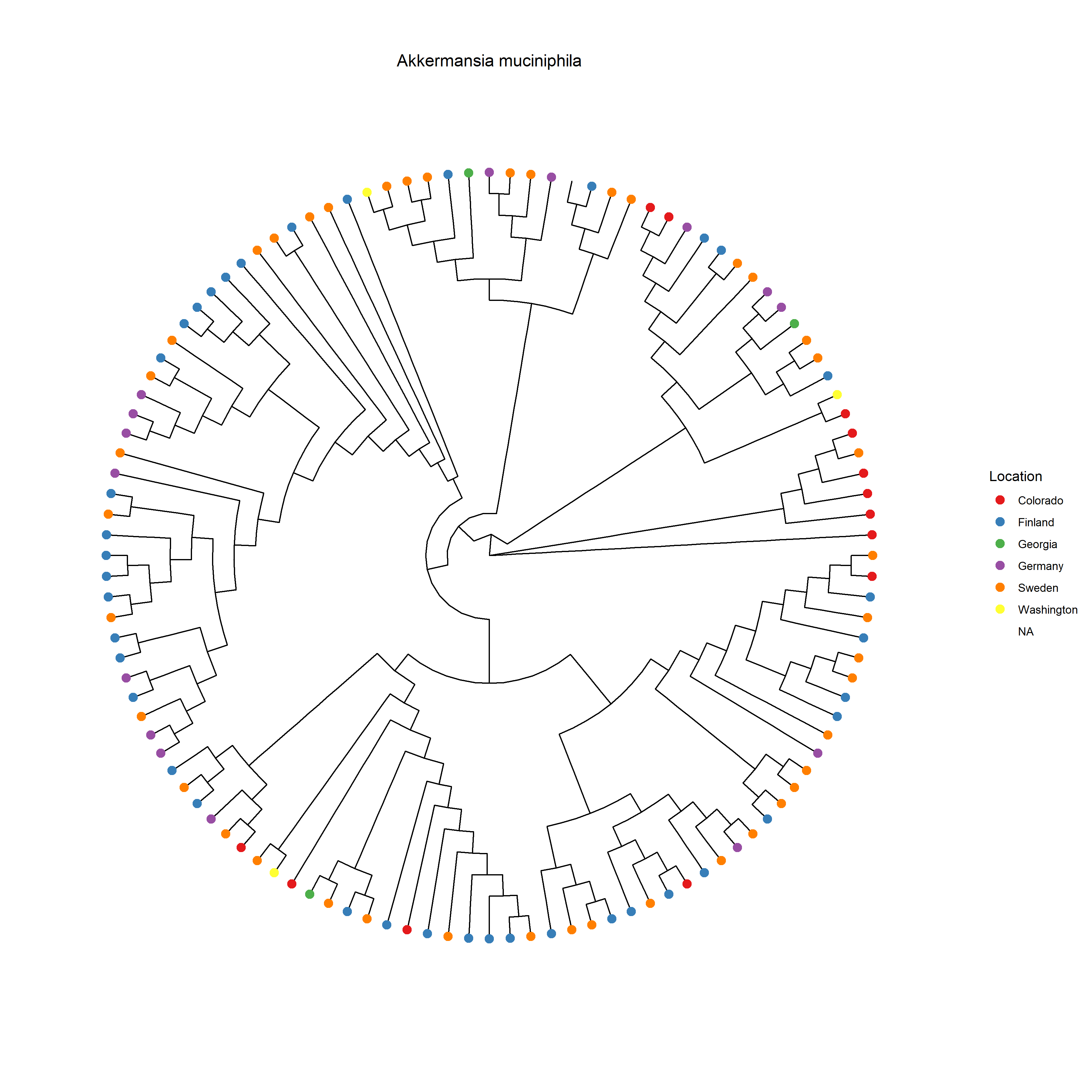

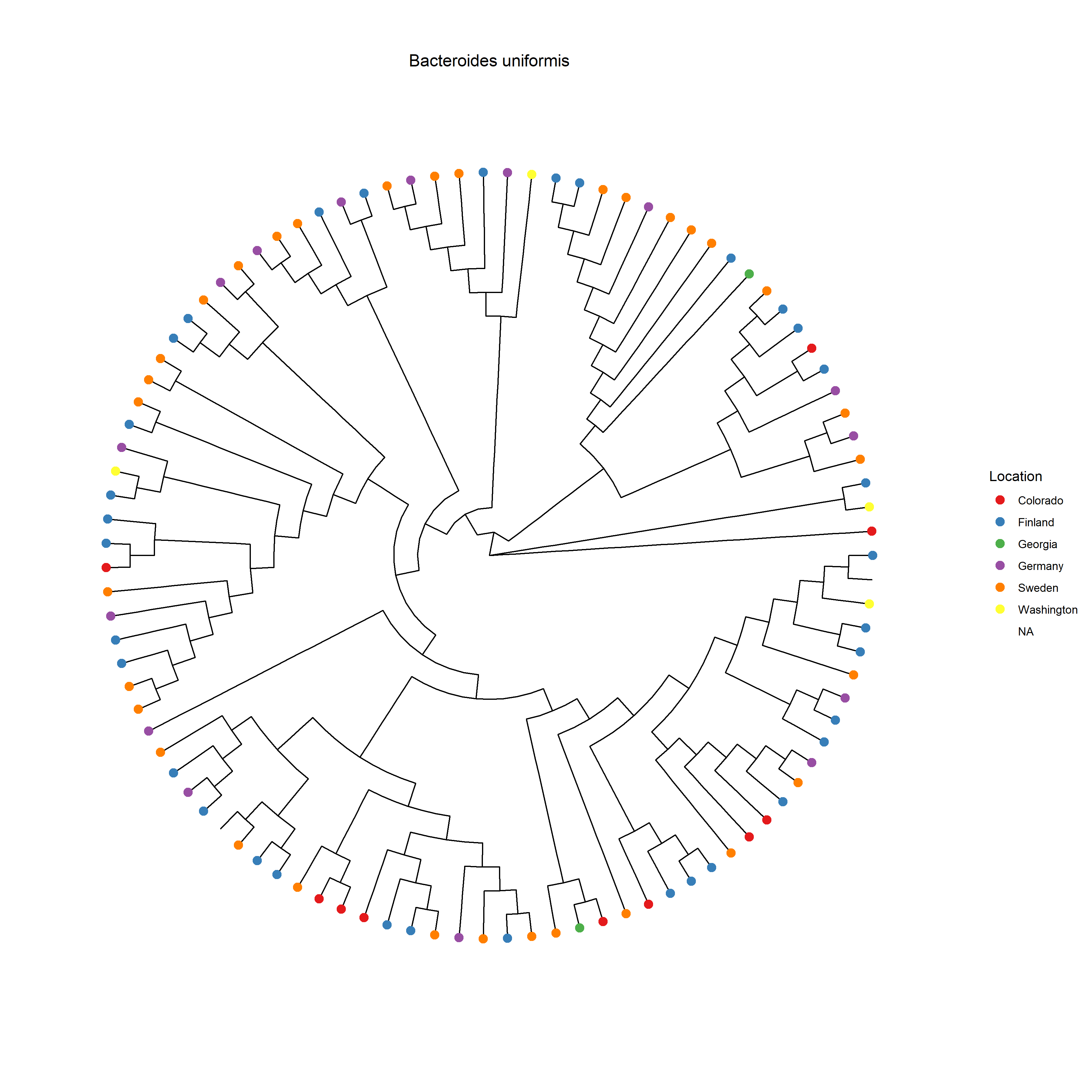

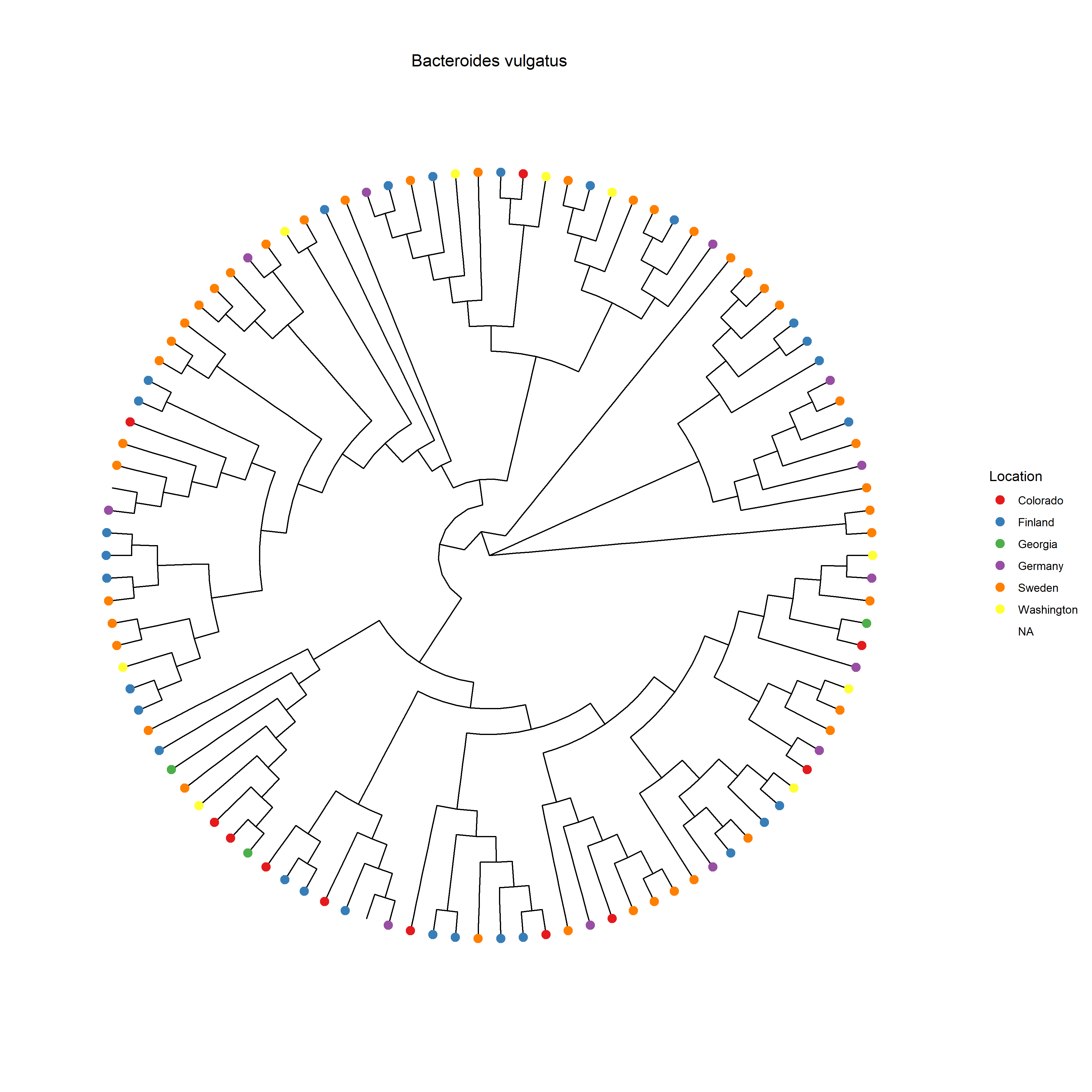

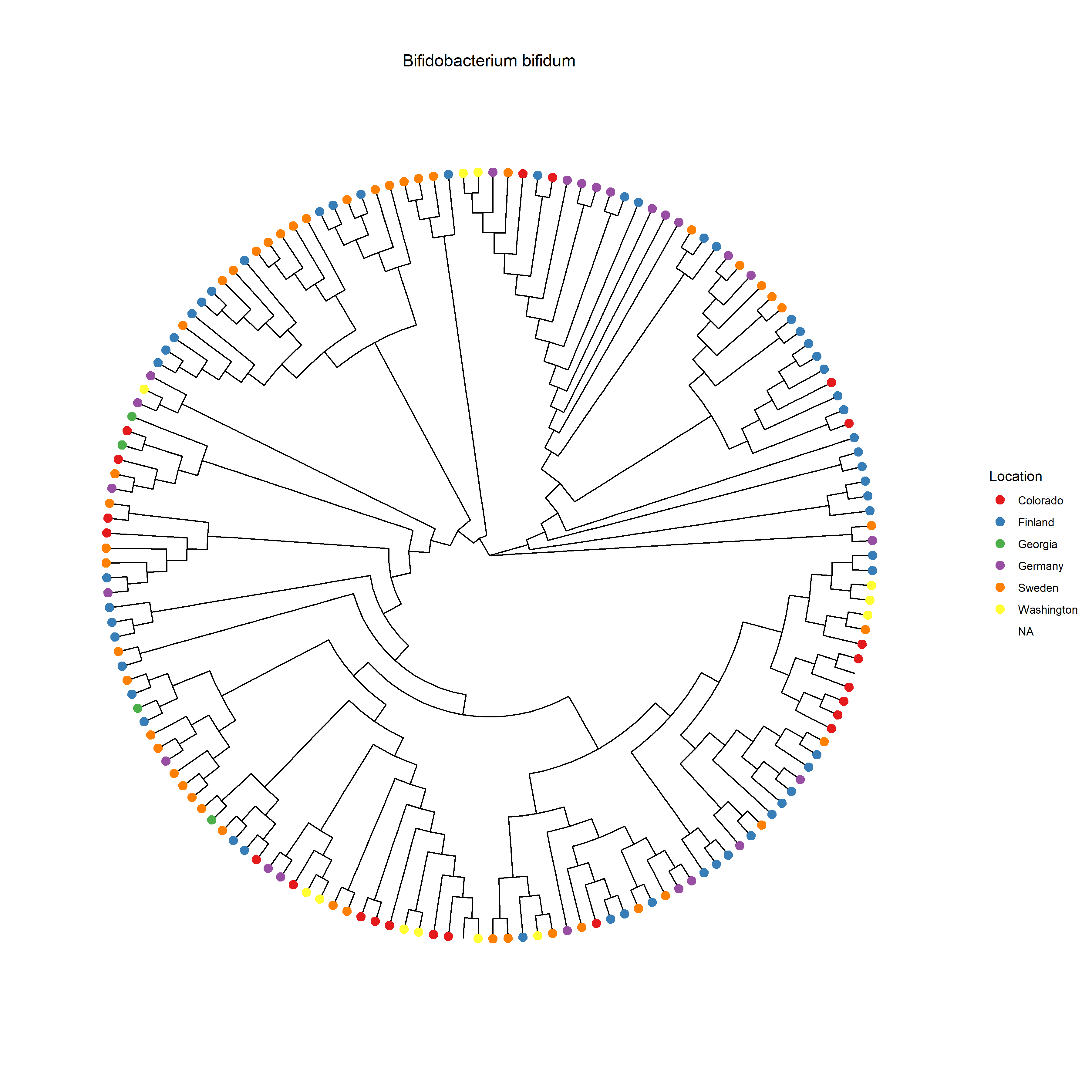

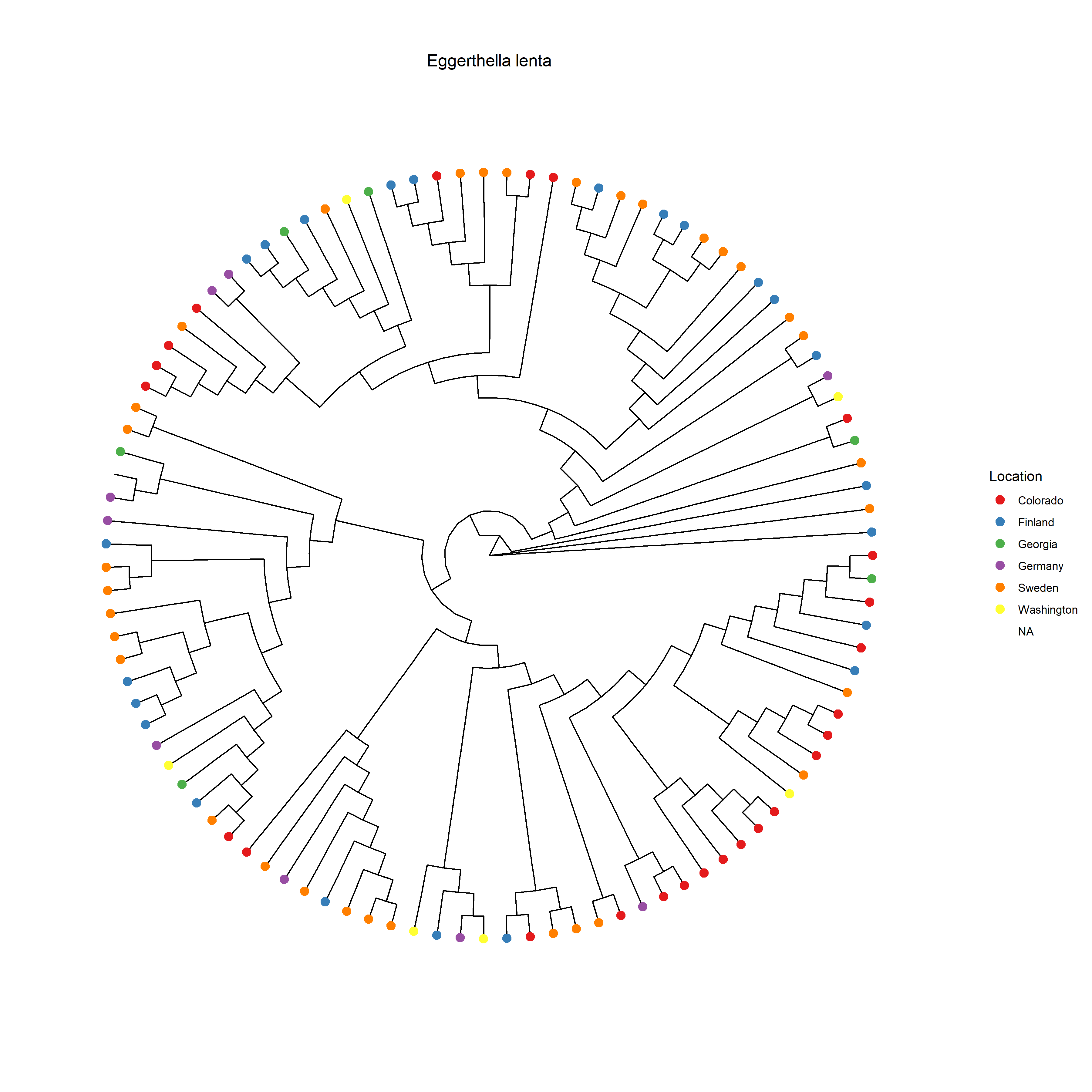

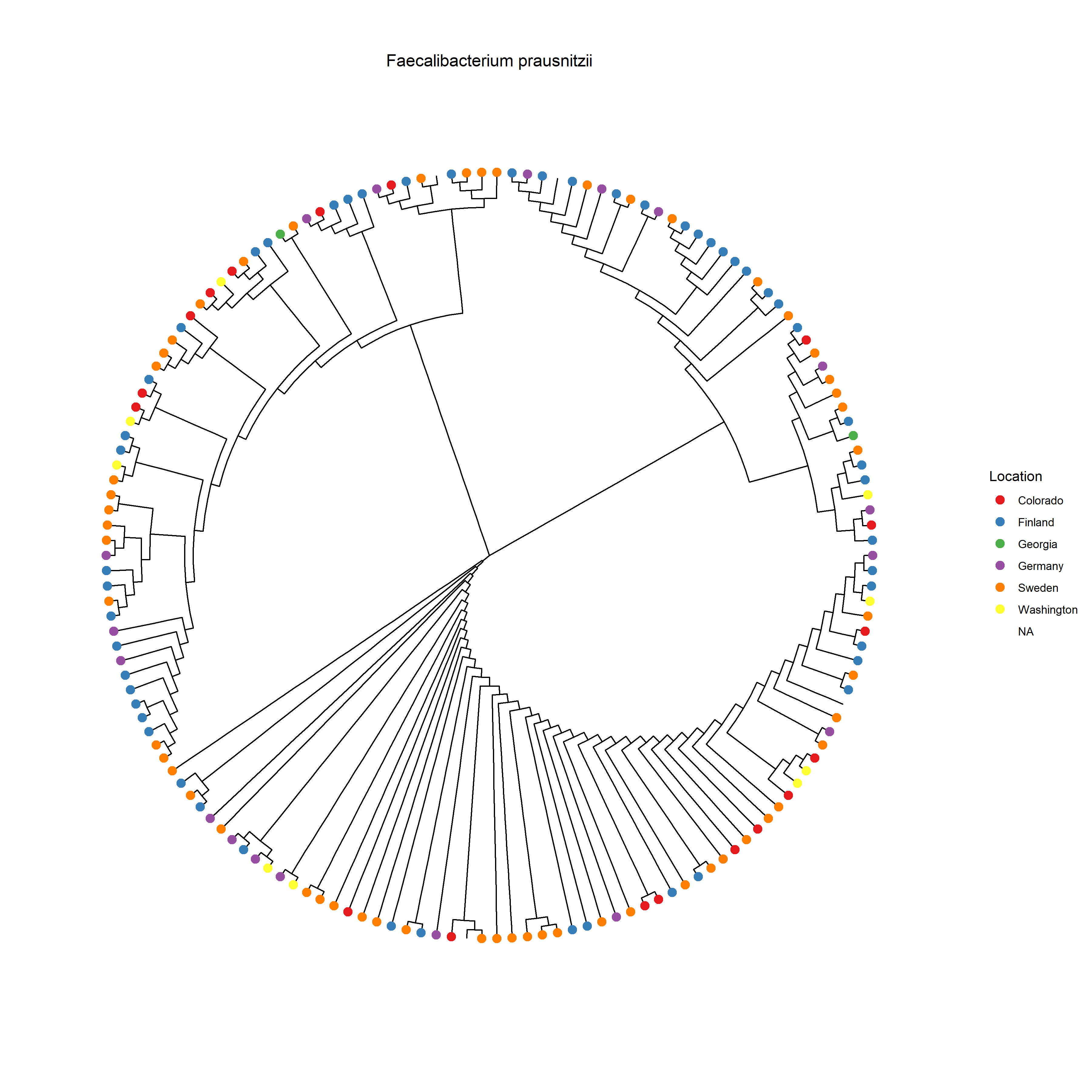


# Supplemental Tables

## Supplemental Table S1. Comparison of LongStrain, MIDAS2, DESMAN, and StrainPhlan4 in precision of SNV calling in community simulation (Gut20) across 20 repetitions

| Species | MIDAS2 | DESMAN | StrainPhlan4 t1 | StrainPhlan4 t2 | StrainPhlan4 t3 | LongStrain primary | LongStrain secondary |
| --- | --- | --- | --- | --- | --- | --- | --- |
| *A. baumannii* | 0.910(0.070) | 0.917(0.086) | 0.987(0.011) | 0.965(0.031) | 0.846(0.163) | 0.986(0.013) | 0.965(0.032) |
| *B. cereus* | 0.907(0.068) | 0.934(0.092) | - | - | - | 0.964(0.035) | 0.892(0.066) |
| *B. fragilis* | 0.918(0.079) | 0.890(0.136) | 0.995(0.009) | 0.986(0.018) | 0.885(0.154) | 0.993(0.008) | 0.959(0.029) |
| *B. adolescentis* | 0.919(0.069) | 0.880(0.118) | 0.993(0.006) | 0.985(0.014) | 0.904(0.127) | 0.988(0.010) | 0.941(0.049) |
| *B. bifidum* | 0.883(0.096) | 0.881(0.129) | 0.996(0.007) | 0.983(0.021) | 0.865(0.154) | 0.994(0.004) | 0.973(0.017) |
| *B. breve* | 0.814(0.061) | 0.712(0.144) | 0.984(0.012) | 0.969(0.015) | 0.884(0.123) | 0.985(0.016) | 0.944(0.046) |
| *B. longum* | 0.873(0.079) | 0.839(0.111) | 0.984(0.008) | 0.963(0.019) | 0.879(0.106) | 0.986(0.012) | 0.945(0.032) |
| *C. beijerinckii* | 0.801(0.101) | - | 0.915(0.051) | 0.871(0.051) | 0.770(0.172) | 0.978(0.023) | 0.958(0.029) |
| *E. faecalis* | 0.912(0.070) | - | 0.996(0.005) | 0.984(0.018) | 0.85(0.164) | 0.995(0.005) | 0.978(0.019) |
| *E. coli* | 0.899(0.073) | 0.919(0.104) | 0.981(0.01) | 0.951(0.019) | 0.844(0.108) | 0.972(0.022) | 0.942(0.029) |
| *H. pylori* | - | - | - | - | - | 0.970(0.030) | 0.947(0.037) |
| *L. gasseri* | 0.907(0.088) | 0.892(0.142) | 0.972(0.020) | 0.957(0.021) | 0.883(0.117) | 0.983(0.017) | 0.948(0.038) |
| *L. monocytogenes* | 0.952(0.066) | 0.926(0.086) | - | - | - | 0.989(0.013) | 0.917(0.059) |
| *N. meningitidis* | 0.926(0.050) | 0.862(0.187) | 0.803(0.102) | 0.640(0.095) | 0.465(0.186) | 0.964(0.032) | 0.975(0.012) |
| *P. aeruginosa* | 0.788(0.160) | 0.901(0.169) | 0.975(0.022) | 0.918(0.051) | 0.671(0.248) | 0.989(0.015) | 0.990(0.007) |
| *R. sphaeroides* | 0.947(0.058) | 0.128(0.131) | 0.985(0.012) | 0.971(0.022) | 0.904(0.124) | 0.990(0.011) | 0.958(0.035) |
| *S. aureus* | 0.936(0.072) | 0.891(0.109) | 0.990(0.007) | 0.979(0.017) | 0.904(0.134) | 0.991(0.009) | 0.973(0.017) |
| *S. epidermidis* | 0.862(0.142) | 0.768(0.191) | 0.980(0.036) | 0.933(0.076) | 0.729(0.286) | 0.983(0.024) | 0.958(0.018) |
| *S. agalactiae* | 0.906(0.081) | 0.819(0.164) | 0.995(0.006) | 0.986(0.014) | 0.899(0.130) | 0.950(0.083) | 0.913(0.056) |
| *S. mutans* | 0.925(0.057) | 0.923(0.099) | 0.990(0.009) | 0.970(0.018) | 0.884(0.124) | 0.993(0.008) | 0.955(0.043) |

## Supplemental Table S2. Comparison of LongStrain, MIDAS2, DESMAN, and StrainPhlan4 in recall of SNV calling in community simulation (Gut20) across 20 repetitions

| Species | MIDAS2 | DESMAN | StrainPhlan4 t1 | StrainPhlan4 t2 | StrainPhlan4 t3 | LongStrain primary | LongStrain secondary |
| --- | --- | --- | --- | --- | --- | --- | --- |
| *A. baumannii* | 0.453(0.050) | 0.097(0.024) | 0.014(0.002) | 0.011(0.002) | 0.010(0.002) | 0.669(0.063) | 0.599(0.089) |
| *B. cereus* | 0.411(0.115) | 0.174(0.084) | - | - | - | 0.336(0.048) | 0.196(0.06) |
| *B. fragilis* | 0.525(0.163) | 0.149(0.117) | 0.024(0.004) | 0.019(0.004) | 0.017(0.004) | 0.793(0.101) | 0.546(0.132) |
| *B. adolescentis* | 0.362(0.110) | 0.101(0.061) | 0.054(0.007) | 0.047(0.006) | 0.044(0.007) | 0.666(0.074) | 0.469(0.127) |
| *B. bifidum* | 0.504(0.236) | 0.098(0.073) | 0.083(0.009) | 0.071(0.012) | 0.066(0.015) | 0.880(0.068) | 0.628(0.164) |
| *B. breve* | 0.466(0.096) | 0.087(0.033) | 0.108(0.013) | 0.094(0.009) | 0.089(0.012) | 0.704(0.103) | 0.540(0.121) |
| *B. longum* | 0.382(0.126) | 0.075(0.035) | 0.081(0.007) | 0.069(0.009) | 0.064(0.010) | 0.578(0.048) | 0.395(0.098) |
| *C. beijerinckii* | 0.353(0.101) | - | 0.042(0.006) | 0.038(0.005) | 0.036(0.006) | 0.699(0.104) | 0.619(0.138) |
| *E. faecalis* | 0.609(0.139) | - | 0.076(0.012) | 0.062(0.010) | 0.056(0.011) | 0.842(0.079) | 0.687(0.125) |
| *E. coli* | 0.444(0.124) | 0.097(0.043) | 0.010(0.001) | 0.008(0.001) | 0.007(0.002) | 0.312(0.038) | 0.204(0.063) |
| *H. pylori* | - | - | - | - | - | 0.345(0.053) | 0.237(0.082) |
| *L. gasseri* | 0.351(0.046) | 0.083(0.038) | 0.060(0.008) | 0.052(0.006) | 0.047(0.006) | 0.652(0.089) | 0.536(0.091) |
| *L. monocytogenes* | 0.333(0.099) | 0.119(0.072) | - | - | - | 0.571(0.061) | 0.345(0.098) |
| *N. meningitidis* | 0.291(0.076) | 0.056(0.016) | 0.015(0.002) | 0.012(0.002) | 0.011(0.002) | 0.535(0.044) | 0.515(0.103) |
| *P. aeruginosa* | 0.677(0.074) | 0.135(0.060) | 0.017(0.003) | 0.012(0.002) | 0.010(0.002) | 0.871(0.065) | 0.801(0.085) |
| *R. sphaeroides* | 0.423(0.149) | 0.008(0.010) | 0.044(0.007) | 0.038(0.007) | 0.035(0.009) | 0.829(0.074) | 0.716(0.142) |
| *S. aureus* | 0.387(0.123) | 0.105(0.055) | 0.022(0.002) | 0.019(0.002) | 0.018(0.004) | 0.781(0.060) | 0.621(0.123) |
| *S. epidermidis* | 0.466(0.207) | 0.179(0.119) | 0.038(0.007) | 0.030(0.009) | 0.027(0.010) | 0.800(0.080) | 0.604(0.173) |
| *S. agalactiae* | 0.480(0.152) | 0.098(0.064) | 0.074(0.009) | 0.065(0.008) | 0.063(0.009) | 0.739(0.178) | 0.430(0.086) |
| *S. mutans* | 0.577(0.167) | 0.142(0.079) | 0.069(0.013) | 0.051(0.013) | 0.044(0.013) | 0.823(0.111) | 0.625(0.138) |

## Supplemental Table S3. Comparison of LongStrain, MIDAS2, DESMAN, and StrainPhlan4 in precision of SNV calling in single-species simulation (*Bifidobacterium breve*) in two scenarios at different depths across 20 repetitions

| Longitudinal Scenario | Sequencing Depth | MIDAS2 | DESMAN | StrainPhlan4 t1 | StrainPhlan4 t2 | StrainPhlan4 t3 | LongStrain primary | LongStrain secondary |
| --- | --- | --- | --- | --- | --- | --- | --- | --- |
| transitional | 5$\times$ | - | - | 0.987(0.005) | 0.925(0.012) | 0.748(0.012) | 0.995(0.001) | 0.982(0.002) |
| stable |  | - | - | 0.973(0.005) | 0.975(0.006) | 0.974(0.007) | 0.997(0.001) | 0.961(0.003) |
| transitional | 10$\times$ | 0.800(0.007) | 0.982(0.014) | 0.992(0.003) | 0.963(0.004) | 0.795(0.010) | 0.997(0.001) | 0.987(0.002) |
| stable |  | 0.991(0.002) | 0.883(0.018) | 0.986(0.002) | 0.988(0.003) | 0.988(0.003) | 0.993(0.001) | 0.957(0.005) |
| transitional | 20$\times$ | 0.911(0.004) | 0.987(0.006) | 0.994(0.002) | 0.978(0.003) | 0.846(0.011) | 0.996(0.001) | 0.992(0.001) |
| stable |  | 0.992(0.001) | 0.984(0.003) | 0.989(0.002) | 0.989(0.003) | 0.989(0.002) | 0.982(0.001) | 0.941(0.003) |

## Supplemental Table S4. Comparison of LongStrain, MIDAS2, DESMAN, and StrainPhlan4 in recall of SNV calling in single-species simulation (*Bifidobacterium breve*) in two scenarios at different depths across 20 repetitions

| Longitudinal Scenario | Sequencing Depth | MIDAS2 | DESMAN | StrainPhlan4 t1 | StrainPhlan4 t2 | StrainPhlan4 t3 | LongStrain primary | LongStrain secondary |
| --- | --- | --- | --- | --- | --- | --- | --- | --- |
| transitional | 5$\times$ | - | - | 0.111(0.004) | 0.088(0.003) | 0.073(0.005) | 0.611(0.006) | 0.511(0.005) |
| stable |  | - | - | 0.102(0.003) | 0.102(0.003) | 0.101(0.005) | 0.602(0.007) | 0.369(0.004) |
| transitional | 10$\times$ | 0.540(0.005) | 0.124(0.002) | 0.119(0.001) | 0.089(0.001) | 0.080(0.001) | 0.777(0.006) | 0.649(0.005) |
| stable |  | 0.407(0.007) | 0.043(0.002) | 0.107(0.002) | 0.107(0.002) | 0.107(0.001) | 0.781(0.004) | 0.523(0.003) |
| transitional | 20$\times$ | 0.631(0.004) | 0.408(0.001) | 0.123(0.001) | 0.086(0.001) | 0.081(0.001) | 0.849(0.003) | 0.704(0.003) |
| stable |  | 0.718(0.003) | 0.287(0.004) | 0.108(0.001) | 0.107(0.001) | 0.108(0.001) | 0.828(0.003) | 0.626(0.004) |

## Supplemental Table S5. The accuracy of the estimated proportion of the primary strain by ConStrains, DESMAN, and LongStrain in single-species simulation (*Bifidobacterium breve*) in two scenarios at different depths across 20 repetitions

| Longitudinal Scenario | Sequencing Depth | ConStrains | | | DESMAN | | |  | LongStrain |  |
| --- | --- | --- | --- | --- | --- | --- | --- | --- | --- | --- |
|  |  | N | MAE | RMSE | N | MAE | RMSE | N | MAE | RMSE |
| transitional | 5$\times$ | - | - | - | - | - | - | 20 | 0.027 | 0.027 |
| stable |  | - | - | - | - | - | - | 20 | 0.007 | 0.008 |
| transitional | 10$\times$ | 20 | 0.069 | 0.109 | 20 | 0.036 | 0.036 | 20 | 0.018 | 0.018 |
| stable |  | 20 | 0.074 | 0.113 | 20 | 0.120 | 0.120 | 20 | 0.003 | 0.004 |
| transitional | 20$\times$ | 20 | 0.052 | 0.084 | 20 | 0.004 | 0.004 | 20 | 0.015 | 0.015 |
| stable |  | 20 | 0.054 | 0.078 | 20 | 0.027 | 0.027 | 20 | 0.002 | 0.002 |

N: the number of successful reports in 20 repetitions of the simulation. MAE: mean absolute error. RMSE: root mean square error.

## Supplemental Table S6. Comparison of LongStrain, MIDAS2, DESMAN, and StrainPhlan4 in precision of SNV calling in single-species simulation (*Bifidobacterium breve*) in two scenarios using references with different ANIs (relative to the representative genome) at sequencing depth of 10$\boldsymbol{\times}$ across 20 repetitions

| Longitudinal Scenario | ANI to representative genome | MIDAS2 | DESMAN | StrainPhlan4 t1 | StrainPhlan4 t2 | StrainPhlan4 t3 | LongStrain primary | LongStrain secondary |
| --- | --- | --- | --- | --- | --- | --- | --- | --- |
| transitional | 99% | 0.849(0.005) | 0.997(0.003) | 0.994(0.002) | 0.961(0.005) | 0.752(0.009) | 0.998(0.001) | 0.995(0.001) |
| stable |  | 0.997(0.001) | 0.917(0.017) | 0.99(0.002) | 0.988(0.002) | 0.990(0.003) | 0.995(0.001) | 0.980(0.001) |
| transitional | 98% | 0.842(0.002) | 0.997(0.003) | 0.996(0.002) | 0.965(0.003) | 0.749(0.007) | 0.999(0.001) | 0.997(0.001) |
| stable |  | 0.996(0.001) | 0.900(0.022) | 0.992(0.002) | 0.992(0.001) | 0.992(0.001) | 0.996(0.001) | 0.987(0.001) |
| transitional | 97% | 0.826(0.003) | 0.998(0.002) | 0.995(0.001) | 0.958(0.003) | 0.749(0.007) | 0.999(0.001) | 0.998(0.001) |
| stable |  | 0.991(0.002) | 0.869(0.021) | 0.989(0.002) | 0.989(0.002) | 0.990(0.001) | 0.996(0.001) | 0.987(0.001) |

## Supplemental Table S7. Comparison of LongStrain, MIDAS2, DESMAN, and StrainPhlan4 in recall of SNV calling in single-species simulation (*Bifidobacterium breve*) in two scenarios using references with different ANIs (relative to the representative genome) at sequencing depth of 10$\boldsymbol{\times}$ across 20 repetitions

| Longitudinal Scenario | ANI to representative genome | MIDAS2 | DESMAN | StrainPhlan4 t1 | StrainPhlan4 t2 | StrainPhlan4 t3 | LongStrain primary | LongStrain secondary |
| --- | --- | --- | --- | --- | --- | --- | --- | --- |
| transitional | 99% | 0.576(0.005) | 0.161(0.002) | 0.096(0.001) | 0.063(0.001) | 0.055(0.001) | 0.846(0.004) | 0.776(0.003) |
| stable |  | 0.18(0.004) | 0.032(0.001) | 0.083(0.002) | 0.083(0.001) | 0.083(0.001) | 0.843(0.003) | 0.657(0.005) |
| transitional | 98% | 0.579(0.003) | 0.179(0.002) | 0.097(0.001) | 0.061(0.001) | 0.053(0.001) | 0.851(0.002) | 0.829(0.002) |
| stable |  | 0.184(0.003) | 0.034(0.002) | 0.083(0.001) | 0.082(0.001) | 0.082(0.001) | 0.832(0.002) | 0.722(0.003) |
| transitional | 97% | 0.502(0.002) | 0.139(0.002) | 0.095(0.001) | 0.061(0.001) | 0.053(0.001) | 0.826(0.002) | 0.808(0.002) |
| stable |  | 0.172(0.002) | 0.027(0.001) | 0.081(0.001) | 0.081(0.001) | 0.081(0.001) | 0.799(0.002) | 0.695(0.002) |

## Supplemental Table S8. The accuracy of the estimated proportion of the primary strain by ConStrains, DESMAN, and LongStrain in single-species simulation (*Bifidobacterium breve*) in two scenarios using references with different ANIs (relative to the representative genome) at sequencing depth of 10$\boldsymbol{\times}$ across 20 repetitions

| Longitudinal Scenario | ANI to representative genome | ConStrains | | | DESMAN | | |  | LongStrain |  |
| --- | --- | --- | --- | --- | --- | --- | --- | --- | --- | --- |
|  |  | N | MAE | RMSE | N | MAE | RMSE | N | MAE | RMSE |
| transitional | 99% | 20 | 0.043 | 0.056 | 20 | 0.043 | 0.043 | 20 | 0.010 | 0.010 |
| stable |  | 20 | 0.058 | 0.091 | 20 | 0.115 | 0.115 | 20 | 0.001 | 0.001 |
| transitional | 98% | 20 | 0.057 | 0.081 | 20 | 0.040 | 0.040 | 20 | 0.010 | 0.010 |
| stable |  | 20 | 0.072 | 0.113 | 20 | 0.134 | 0.134 | 20 | 0.001 | 0.001 |
| transitional | 97% | 20 | 0.044 | 0.056 | 20 | 0.045 | 0.045 | 20 | 0.012 | 0.012 |
| stable |  | 20 | 0.073 | 0.114 | 20 | 0.168 | 0.168 | 20 | 0.002 | 0.002 |

N: the number of successful reports in 20 repetitions of the simulation. MAE: mean absolute error. RMSE: root mean square error.

## Supplemental Table S9. The accuracy of the estimated proportion of the primary strain by ConStrains, DESMAN, and LongStrain in single-species simulation (*Bifidobacterium breve*) in three-strain scenario at depth of 10$\boldsymbol{\times}$

| Longitudinal Scenario | ConStrains | | DESMAN | | LongStrain | |
| --- | --- | --- | --- | --- | --- | --- |
|  | MAE | RMSE | MAE | RMSE | MAE | RMSE |
| [8:1:1, 5:4:1, 2:7:1] | 0.119 | 0.132 | 0.034 | 0.034 | 0.100 | 0.100 |
| [7:2:1, 7:2:1, 7:2:1] | 0.112 | 0.125 | 0.320 | 0.321 | 0.063 | 0.063 |
| [9:1:0, 6:0:4, 3:7:0] | 0.171 | 0.192 | 0.051 | 0.051 | 0.076 | 0.076 |
| [8:2:0, 8:0:2, 0:8:2] | 0.068 | 0.075 | 0.018 | 0.019 | 0.096 | 0.096 |

MAE: mean absolute error. RMSE: root mean square error.

## Supplemental Table S10. Association test of the log2-ratio (primary strain proportion/secondary strain proportion) and the birth mode (caesarian or vaginal) by the linear mixed model

| Species | Month | p-value FDR | BirthMode-Vaginal | p-value FDR | Gender-Male | p-value FDR | Interaction-Month-BirthMode | p-value FDR |
| --- | --- | --- | --- | --- | --- | --- | --- | --- |
| ***Akkermansia muciniphila*** | **0.218099** | **0.117597** | **8.115809** | **0.014688** | **-0.25837** | **0.960762** | **-0.45919** | **0.019681** |
| *Anaerostipes hadrus* | -0.07583 | 0.117597 | -1.45104 | 0.333477 | -0.18694 | 0.933131 | 0.089497 | 0.130442 |
| *Bacteroides caccae* | -0.13064 | 0.760105 | -4.15278 | 0.620057 | 0.432207 | 0.925338 | 0.254275 | 0.406928 |
| *Bacteroides fragilis* | 0.133718 | 0.055902 | 1.473342 | 0.388051 | -0.4426 | 0.757659 | -0.14297 | 0.067735 |
| *Bacteroides sp. A1C1* | -0.00061 | 0.993257 | -0.7143 | 0.86315 | -0.49733 | 0.925338 | -0.08744 | 0.462563 |
| ***Bacteroides thetaiotaomicron*** | **0.205937** | **0.00506** | **3.930116** | **0.015161** | **-0.77379** | **0.369791** | **-0.20369** | **0.019681** |
| *Bacteroides uniformis* | -0.00268 | 0.993257 | -1.6643 | 0.620057 | -0.3704 | 0.925338 | 0.031322 | 0.867946 |
| *Bacteroides vulgatus* | 0.027204 | 0.854146 | 0.02092 | 0.984408 | 0.100716 | 0.960762 | -0.09528 | 0.210947 |
| *Bifidobacterium adolescentis* | -0.07387 | 0.524917 | -1.33927 | 0.647148 | -0.25639 | 0.941162 | 0.170396 | 0.07128 |
| *Bifidobacterium bifidum* | -0.00852 | 0.993257 | -0.33777 | 0.86315 | 0.078204 | 0.960762 | 0.013127 | 0.874599 |
| ***Bifidobacterium breve*** | **-0.17011** | **8.63E-06** | **-2.37154** | **0.000993** | **0.401911** | **0.694704** | **0.235589** | **5.79E-08** |
| *Bifidobacterium catenulatum* | 0.168333 | 0.03257 | 2.840976 | 0.206627 | -0.02918 | 0.967586 | -0.16119 | 0.07128 |
| *Bifidobacterium dentium* | -0.01508 | 0.993257 | -0.67016 | 0.897126 | -0.05799 | 0.967586 | 0.076817 | 0.817795 |
| *Bifidobacterium longum* | -0.07316 | 0.038009 | 0.356732 | 0.756883 | -0.16717 | 0.925338 | -0.07297 | 0.07128 |
| *Bifidobacterium pseudocatenulatum* | 0.000444 | 0.993257 | 0.784888 | 0.756883 | -0.14715 | 0.960762 | 0.009951 | 0.874599 |
| *Eggerthella enta* | -0.11553 | 0.003729 | 0.771237 | 0.658904 | -1.07928 | 0.369791 | -0.0398 | 0.480143 |
| *Enterococcus faecalis* | 0.317545 | 0.727881 | -3.10844 | 0.647148 | -0.60215 | 0.925338 | 0.124345 | 0.874599 |
| *Escherichia coli* | -0.08493 | 0.066405 | 0.185967 | 0.897126 | -0.56877 | 0.369791 | -0.05202 | 0.41979 |
| ***Faecalibacterium prausnitzii*** | **0.020557** | **0.727881** | **0.762205** | **0.453186** | **-0.54935** | **0.363644** | **-0.07973** | **0.034136** |
| *Flavonifractor plautii* | 0.069626 | 0.393844 | -0.03313 | 0.984408 | -0.81294 | 0.407739 | -0.04302 | 0.746763 |
| *Lachnospiraceae bacterium GAM79* | 0.022803 | 0.855774 | -0.68084 | 0.785211 | -0.05275 | 0.967586 | 0.00934 | 0.874599 |
| *Roseburia hominis* | -0.0315 | 0.760105 | -2.14825 | 0.294221 | 0.371293 | 0.925338 | 0.097505 | 0.210947 |
| ***Roseburia intestinalis*** | **0.014513** | **0.921798** | **2.34299** | **0.046731** | **-0.80962** | **0.369791** | **-0.13534** | **0.034136** |
| *Ruthenibacterium lactatiformans* | -0.00422 | 0.993257 | 0.824045 | 0.756883 | -1.60816 | 0.343527 | -0.06804 | 0.41979 |
| *Streptococcus thermophilus* | 0.094386 | 0.122189 | -0.21545 | 0.903611 | 0.253395 | 0.925338 | 0.037905 | 0.767442 |
| *Veillonella parvula* | -0.18104 | 0.03257 | 0.190067 | 0.903611 | -0.60435 | 0.407739 | 0.012966 | 0.874599 |

The linear mixed model: $\log_{2} Ratio \sim\beta_{0}+\beta_{1}Month+\beta_{2}BirthModel+\beta_{3}Month:BirthModel+\beta_{4}Gender+\left( 1|ID \right)$

| Species | Primary Strain | | | Secondary Strain | | | ANI^*^ |
| --- | --- | --- | --- | --- | --- | --- | --- |
|  | Genome ID | Completeness | ANI to representative genome | Genome ID | Completeness | ANI to representative genome |  |
| *A. baumannii* | GCF_000021245.2 | 98.94% | 98.61% | GCF_000018445.1 | 98.66% | 98.69% | 98.55% |
| *B. cereus* | GCF_001721145.1 | 99.06% | 99.35% | GCF_002504205.1 | 98.90% | 99.40% | 99.42% |
| *B. fragilis* | GCF_000009925.1 | 98.73% | 99.55% | GCF_000210835.1 | 98.73% | 99.54% | 99.50% |
| *B. adolescentis* | GCF_000817995.1 | 96.78% | 99.18% | GCF_000737885.1 | 97.61% | 99.19% | 99.21% |
| *B. bifidum* | GCF_000164965.1 | 98.04% | 99.36% | GCF_002845845.1 | 98.38% | 99.26% | 99.29% |
| *B. breve* | GCF_000213865.1 | 99.9% | 99.35% | GCF_000568955.1 | 99.90% | 99.28% | 99.27% |
| *B. longum* | GCF_000007525.1 | 99.78% | 99.40% | GCF_000008945.1 | 99.57% | 99.36% | 99.34% |
| *C. beijerinckii* | GCF_000506785.4 | 93.11% | 98.91% | GCF_002003345.1 | 92.59% | 98.46% | 98.36% |
| *E. faecalis* | GCF_000007785.1 | 99.66% | 99.27% | GCF_000172575.2 | 99.66% | 99.32% | 99.30% |
| *E. coli* | GCF_000026545.1 | 99.24% | 99.01% | GCF_000010485.1 | 98.70% | 99.04% | 98.86% |
| *H. pylori* | GCF_000091345.1 | 99.47% | 97.12% | GCF_000196755.1 | 98.81% | 97.05% | 96.77% |
| *L. gasseri* | GCF_000143645.1 | 97.9% | 99.15% | GCF_002158885.1 | 95.96% | 99.17% | 99.02% |
| *L. monocytogenes* | GCF_000022925.1 | 99.23% | 99.24% | GCF_000196035.1 | 98.72% | 99.25% | 99.30% |
| *N. meningitidis* | GCF_000009105.1 | 98.55% | 99.45% | GCF_000009465.1 | 99.41% | 98.37% | 98.31% |
| *P. aeruginosa* | GCF_000006765.1 | 99.39% | 99.56% | GCF_000014625.1 | 98.95% | 99.13% | 99.11% |
| *R. sphaeroides* | GCF_000021005.1 | 99.03% | 98.27% | GCF_000015985.1 | 98.00% | 98.62% | 98.19% |
| *S. aureus* | GCF_000009005.1 | 97.60% | 98.70% | GCF_000009585.1 | 98.20% | 98.63% | 98.49% |
| *S. epidermidis* | GCF_000007645.1 | 99.69% | 99.75% | GCF_000011925.1 | 99.76% | 99.54% | 99.50% |
| *S. agalactiae* | GCF_000007265.1 | 99.78% | 99.39% | GCF_000196055.1 | 99.29% | 99.43% | 99.61% |
| *S. mutans* | GCF_000091645.1 | 99.53% | 99.40% | GCF_000007465.2 | 99.66% | 99.50% | 99.36% |

## Supplemental Table S11. Detailed information of strain genomes used in community simulation

*: ANI between the genome of primary strain and the genome of secondary strain

| Species | mean | SD |
| --- | --- | --- |
| *A. baumannii* | 0.820 | 0.007 |
| *B. cereus* | 0.582 | 0.013 |
| *B. fragilis* | 0.839 | 0.004 |
| *B. adolescentis* | 0.809 | 0.008 |
| *B. bifidum* | 0.915 | 0.003 |
| *B. breve* | 0.881 | 0.009 |
| *B. longum* | 0.687 | 0.010 |
| *C. beijerinckii* | 0.856 | 0.006 |
| *E. faecalis* | 0.875 | 0.004 |
| *E. coli* | 0.515 | 0.020 |
| *H. pylori* | 0.696 | 0.026 |
| *L. gasseri* | 0.844 | 0.008 |
| *L. monocytogenes* | 0.904 | 0.005 |
| *N. meningitidis* | 0.882 | 0.010 |
| *P. aeruginosa* | 0.938 | 0.003 |
| *R. sphaeroides* | 0.869 | 0.007 |
| *S. aureus* | 0.890 | 0.006 |
| *S. epidermidis* | 0.945 | 0.002 |
| *S. agalactiae* | 0.774 | 0.001 |
| *S. mutans* | 0.930 | 0.009 |

## Supplemental Table S12. Horizontal genome coverage for each species in community simulation
